# Population assessment using multivariate time‐series analysis: A case study of rockfishes in Puget Sound

**DOI:** 10.1002/ece3.2901

**Published:** 2017-03-21

**Authors:** Nick Tolimieri, Elizabeth E. Holmes, Gregory D. Williams, Robert Pacunski, Dayv Lowry

**Affiliations:** ^1^ Conservation Biology DivisionNorthwest Fisheries Science CenterNational Marine Fisheries ServiceNational Oceanic and Atmospheric AdministrationSeattleWAUSA; ^2^ Pacific States Marine Fisheries Commission, Under Contract to Northwest Fisheries Science CenterNational Marine Fisheries ServiceNational Oceanic and Atmospheric AdministrationSeattleWAUSA; ^3^ Marine Fish Science UnitFish Management DivisionWashington Department of Fish and WildlifeMill CreekWAUSA; ^4^ Marine Fish Science UnitFish Management DivisionWashington Department of Fish and WildlifeOlympiaWAUSA

**Keywords:** data‐limited, Endangered Species Act, multivariate autoregressive state‐space models, population viability analysis, risk assessment, rockfishes, *Sebastes*, trend analysis

## Abstract

Estimating a population's growth rate and year‐to‐year variance is a key component of population viability analysis (PVA). However, standard PVA methods require time series of counts obtained using consistent survey methods over many years. In addition, it can be difficult to separate observation and process variance, which is critical for PVA. Time‐series analysis performed with multivariate autoregressive state‐space (MARSS) models is a flexible statistical framework that allows one to address many of these limitations. MARSS models allow one to combine surveys with different gears and across different sites for estimation of PVA parameters, and to implement replication, which reduces the variance‐separation problem and maximizes informational input for mean trend estimation. Even data that are fragmented with unknown error levels can be accommodated. We present a practical case study that illustrates MARSS analysis steps: data choice, model set‐up, model selection, and parameter estimation. Our case study is an analysis of the long‐term trends of rockfish in Puget Sound, Washington, based on citizen science scuba surveys, a fishery‐independent trawl survey, and recreational fishery surveys affected by bag‐limit reductions. The best‐supported models indicated that the recreational and trawl surveys tracked different, temporally independent assemblages that declined at similar rates (an average of −3.8% to −3.9% per year). The scuba survey tracked a separate increasing and temporally independent assemblage (an average of 4.1% per year). Three rockfishes (bocaccio, canary, and yelloweye) are listed in Puget Sound under the US Endangered Species Act (ESA). These species are associated with deep water, which the recreational and trawl surveys sample better than the scuba survey. All three ESA‐listed rockfishes declined as a proportion of recreational catch between the 1970s and 2010s, suggesting that they experienced similar or more severe reductions in abundance than the 3.8–3.9% per year declines that were estimated for rockfish populations sampled by the recreational and trawl surveys.

## 1 Introduction

Population growth rate estimates are critical to many assessment protocols for evaluating the status of species and populations thought to be at risk of extinction, such as those listed in the IUCN Red List Categories and Criteria and the US Endangered Species Act (ESA) (Andelman, Groves, & Regan, 2004; Morris & Doak, 2002; Rueda‐Cediel, Anderson, Regan, Franklin, & Regan, 2015). Count‐based PVA is a standard method used for estimating long‐term average population growth rate and associated year‐to‐year variability (Beissinger, 2002; Dennis, Munholland, & Scott, 1991; Morris & Doak, 2002). Unfortunately, count‐based PVA requires a continuous time series from surveys with consistent methodology. The available data for many species of conservation interest do not meet this standard (Butchart & Bird, 2010; Smith et al., 2009). Frequently, these “data‐poor” species are given an ambiguous status, such as “species of concern” under the ESA, or are designated as “data‐deficient” on the IUCN Red List (Butchart & Bird, 2010; Morais et al., 2013). In these cases, species must await the availability of further research before receiving a clear status or the recovery actions associated with a listing decision (Anderson, Lee, & Levin, 2013). A species may be given a “data‐deficient” designation when data do exist, but are fragmented into multiple short or discontinuous datasets from disparate sources and surveys. In this study, we show how multivariate autoregressive state‐space (MARSS) models can help circumvent some of these dataset challenges.

Classic count‐based PVA (sensu Dennis et al., 1991) uses a univariate autoregressive process to model underlying population abundance trajectories. Multivariate autoregressive (MAR) time‐series models are a multivariate version of the same model that allow one to model multiple population processes with structure or interactions. MAR models have been widely used to examine food web dynamics, species interactions, metapopulation structure, and community stability (Hampton et al., 2013; Ives, Dennis, Cottingham, & Carpenter, 2003; Thibaut, Connolly, & Sweatman, 2012; Ward et al., 2010). A MARSS model is a state‐space version of a MAR model with separate state and observation components, both of which are multivariate. The state is what we aim to estimate (e.g., abundance trajectory), and the data are observations of this state. MARSS models have a well‐established statistical framework for analyzing multivariate time‐series data (cf. Harvey, 1990; and many other textbooks; Shumway & Stoffer, 2010). These models have been used in a variety of ecological and fisheries applications, including analysis of age‐structured populations (Buckland, Newman, Thomas, & Koesters, 2004), analysis of spatial structure in populations (Ward et al., 2010), and factor analysis for large fishery datasets (Zuur, Tuck, & Bailey, 2003). We use MARSS models to tackle the problem of estimating PVA parameters from multivariate data sets, and show how these models can be used to implement replication, solving a difficult variance separation problem, and to test data supporting different underlying population structures, which can affect PVA parameter estimates and the interpretation of those estimates.

Standard PVA, even if conducted with a state‐space model, requires a single time series of data. This requirement limits our ability to systematically combine information from multiple sources and to evaluate the spatial structure of populations. Separate state and observation components combined with a multivariate structure allow MARSS models to treat multiple time series across different, possibly non‐overlapping, year ranges as observations of a single underlying population trajectory, wherein the trajectory reflects an index of the yearly population abundance. Each observation type can be allowed to have a unique abundance scale (i.e., catchability) and level of observation error. Additionally, the state‐space structure and estimation via a Kalman Filter allow missing values to be easily handled (Holmes, Ward, & Scheuerell, 2014).

The MARSS framework also allows us to formally test how well data support structure within both the population process and the observation process. For example, in our rockfish analysis that follows, we test models that treat (1) all recreational and trawl surveys as observing a single abundance trajectory corresponding to a rockfish assemblage with a wide depth range, and (2) all shallow‐water scuba surveys as observations of a separate trajectory. Similarly, MARSS models can be used to evaluate the support for spatial structure (e.g., regional subdivisions) in the underlying abundance trajectories and the support for spatial temporal independence (Hinrichsen & Holmes, 2009; Ward et al., 2010). This utility is important for statistical reasons when estimating population growth rates, but also has implications for population viability. Regions with temporally independent dynamics can buffer risk, and spatial structure affects management decisions and planning (Beaudreau & Whitney, 2016; Hilborn, Quinn, Schindler, & Rogers, 2003; Schindler, Armstrong, & Reed, 2015).

**Figure 1:**
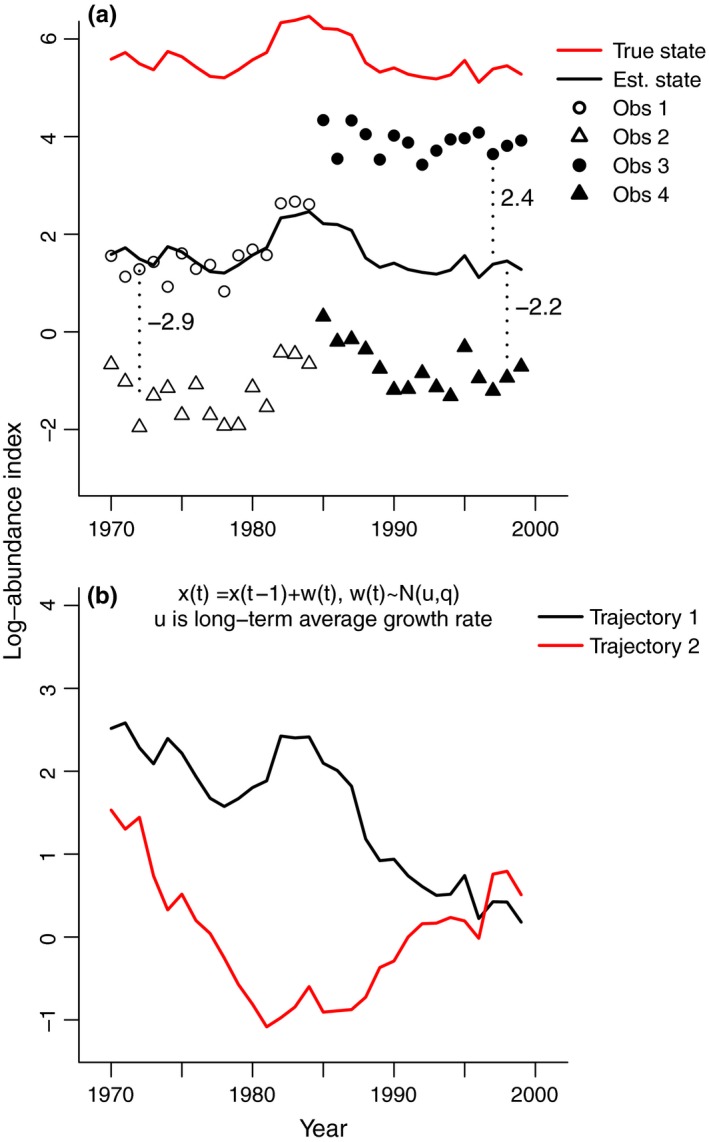
Illustration of the structure of a multivariate autoregressive state‐space (MARSS)‐based population viability analysis (PVA). (a) Four surveys (Obs 1–4) with one true population trajectory (red line) and an estimated trajectory (scaled estimate, black line). The true population trajectory is “hidden” (i.e., not directly observable). The four different time series (surveys) follow the hidden population trajectory but with different scaling because each is a somewhat different index survey. Numbers on the figure indicate *a* (scaling up or down) for the survey data. Only three of the scaling factors can be estimated. One scaling factor is set to zero and the estimate for the population trajectory is scaled to that survey. The scaling factors for the other surveys can be estimated because they are all assumed to be observing the same population (black line). Although estimation is improved if the segments overlap, the model can still estimate the black line, and parameters associated with it, when there are gaps between segments as long as the segments are not too short. Replication by way of multiple observations at different sites or with different surveys can enhance the ability of the model to estimate population trajectory substantially. (b) An example of a MARSS model with the same long‐term population growth rate *u* but two different trajectories (states) which covary

**Figure 2:**
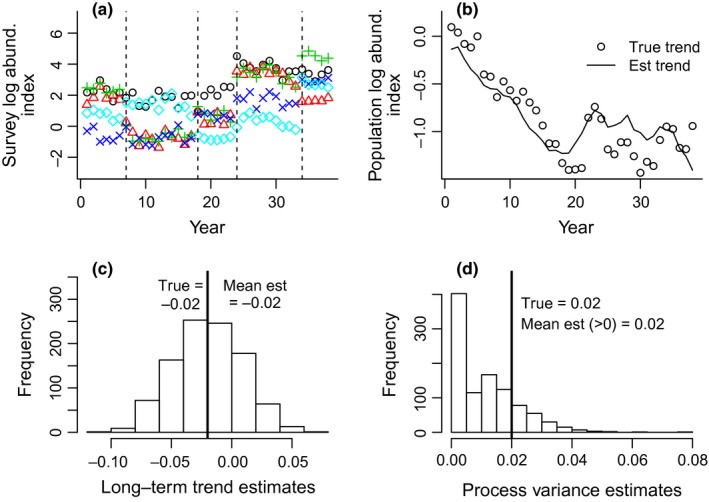
Results of the simulation study of estimation of the population growth and process error for data structured similar to the Recreational Fishery (Rec) survey in the North Puget Sound (NPS). The data are divided into segments of 6, 11, 6, 10, and 5 years long. There are three spatial replicates [marine catch areas (MCAs)]. In this simulation, there is one population trajectory representing the rockfish assemblage surveyed by the Rec survey in NPS. The true population is declining 2.0% per year on average in the simulation; process variance was set to 0.02. For this simulation, the scaling factors were chosen to increase with each successive segment, creating the illusion of an increasing trend. One thousand datasets and population trajectories were generated; long‐term population growth rate and process variance were estimated for each. (a) Example of the true population trajectory and estimated population trajectory for one dataset. (b) Histogram of the 1000 long‐term population growth rate estimates showing that they are unbiased (i.e., non‐zero). (c) Histogram of the process variance estimates. A feature of state‐space models is that there can be a likelihood maximum with one of the variances at zero (degenerate model). The interior (non‐zero) variance estimates are unbiased. In practice, if a degenerate estimate occurs, one examines the likelihood surface to find the interior local maximum where all variances are non‐zero

A key component of a count‐based PVA is estimation of the process variance, which represents real fluctuations in population abundance due to stochastic environmental variation (e.g., variable recruitment due to climate). In contrast, observation variance results from errors in our observation of the hidden, true abundance. State‐space models allow one to partition the variance in time‐series data into process and observation variances by allowing the structure to be modeled in both the population and observation processes (Holmes, 2001; Holmes, Ward, & Wills, 2012; Ward et al., 2010). This partitioning is essential for unbiased forecasting of extinction (Holmes, Sabo, Viscido, & Fagan, 2007; Ward et al., 2010), correctly computing the uncertainty in population trend estimates (Humbert, Mills, Horne, & Dennis, 2009), estimating the strength of population regulation (Dennis, Ponciano, Lele, Taper, & Staples, 2006), and understanding the environmental drivers of population fluctuations (Ahrestani, Hebblewhite, & Post, 2013; Linden & Knape, 2009). Separation of the process and observation variance can be difficult with univariate time series (Holmes, 2004), and the problem can be severe if one is estimating density dependence in the model (Dennis et al., 2006; Knape, 2008). The incorporation of multiple observations (replication) of abundance in MARSS models reduces this separation problem substantially (Dennis, Ponciano, & Taper, 2010; Knape, Besbeas, & De Valpine, 2013) and allows one to estimate the critical process variance parameter. Figure 1a illustrates the structure of data‐versus‐population trajectory relationship in a MARSS framework and shows an example of replicated and fragmented data. Figure 2 shows simulation results from MARSS models applied to simulated datasets similar to the North Puget Sound (NPS) recreational data used in our study. Our rockfish case study illustrates a more complex application of MARSS models wherein replication takes multiple forms across space, changes in fishing regulations, and different survey types. We show how the MARSS framework allows one to include multiple types of replication in order to maximize the power to separate the variances and estimate trends.

Our case study concerns the estimation of the long‐term growth rate and process variance for a multi‐species rockfish (*Sebastes* spp.) assemblage in the Puget Sound in Washington State, USA. This diverse assemblage has experienced several decades of fishing (Drake et al., 2010; Tonnes et al., 2016; Williams, Levin, & Palsson, 2010) and includes three species whose distinct population segments in the Puget Sound were listed in 2010 under the US ESA (75 FR 22276): bocaccio *Sebastes paucispinis* (endangered), canary rockfish *S. pinniger* (threatened), and yelloweye rockfish *S. ruberrimus* (threatened). Time‐series data were available from three very different survey types that varied in duration, completeness, and geographic extent: a recreational fishery survey, a citizen science scuba survey, and a fishery‐independent trawl survey. None of the three data sets alone was amenable to univariate, count‐based PVA methods. The longest time series (the recreational survey) spanned multiple management regimes with potentially different catch‐per‐unit effort (CPUE), and all three surveys had spatial structure and data gaps. Here, we employ MARSS models to use all three datasets together to estimate key PVA parameters and to evaluate data support for spatial structure in the process and observational components. We use information on changes over time in the species composition of the rockfish assemblage to infer long‐term trends (since 1977) of the three listed rockfish species.

## 2 Materials and Methods

As part of the original ESA‐listing for bocaccio, canary, and yelloweye (Drake et al., 2010), we used MARSS models to analyze historical changes in the abundance of “total rockfish” (summed abundance of all rockfish) because there were few observations of the focal species, some concern about species identification, and many taxa had been lumped into a “rockfish” category in the surveys. Thus, individual species analyses were not possible. For this study, we add new data extending the analyses from 2008 to 2014 and change the spatial resolution of the data used from two large regions (north and south Puget Sound) to nine smaller marine catch areas (MCAs) (Data S1). The statistical analysis in this study also differs significantly from that in the study by Drake et al. (2010), whose primary goal was to develop a trend estimate because the study was part of a specific ESA listing analysis that required an estimate of historical trends. Report from Drake et al. was not written to provide the background that would be needed for applying similar approaches. Here, we present the approach for a general audience interested in count‐based PVA using multivariate methods. We show how model selection, combined with survey knowledge, can be used to choose the structure of the model and to evaluate spatial structure in the population, both key considerations when embarking on a multivariate PVA. We also illustrate the use of simulations for the evaluation of parameter estimation performance (Figure 2).

### 2.1 Rockfishes in Puget Sound

**Figure 3:**
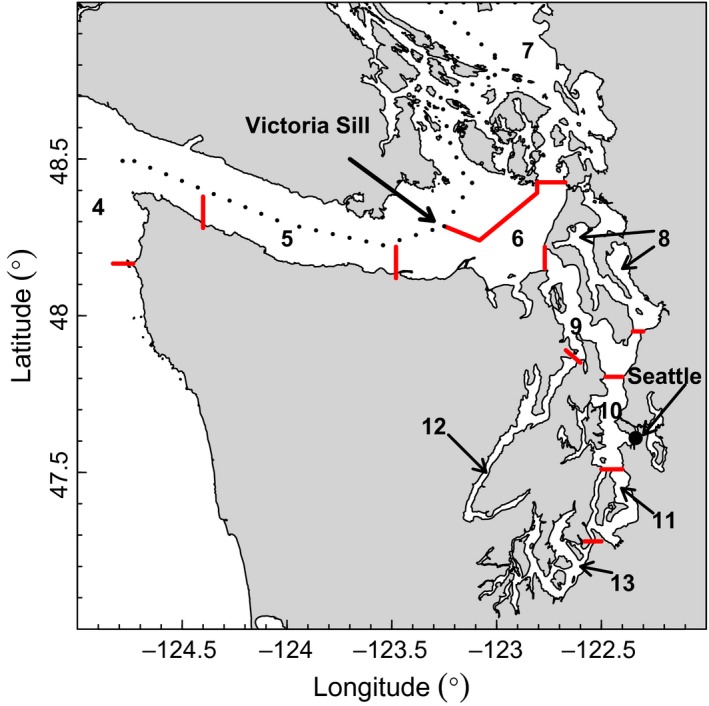
Marine catch areas (MCAs) for Washington Department of Fish and Wildlife (WDFW) recreational catch data. Data from North Puget Sound [(NPS) MCAs 5–7] and Puget Sound Proper [PSP (MCAs 8–13)] were used in the analyses. The major regional water masses are NPS (MCAs 5–7), Whidbey Basin (MCA 8), Main Basin (MCAs 9–11, 13), and Hood Canal (MCA 12). The dashed line indicates the US.–Canadian border. Red lines indicate MCA boundaries

The *Sebastes* spp. are a diverse taxon of demersal and mid‐water marine fishes with 100+ species worldwide, including over 70 in the northeastern Pacific (Love, Yoklavich, & Thorsteinson, 2002; Williams et al., 2010). Twenty‐eight *Sebastes* species have been recorded in the Salish Sea (Pietsch & Orr, 2015), of which Puget Sound is a part. All rockfishes share the characteristics of internal brooding, pelagic larval duration of several months, settlement to shallow vegetated habits, slow maturation, and long‐life spans (Love et al., 2002). Many species are most commonly found in rocky habitats, which are more abundant in northern than in southern areas of Puget Sound (Williams et al., 2010). Within Puget Sound (Figure 3, Drake et al., 2010), sills, freshwater inputs, and current patterns create five hydrographic basins. These characteristics have the potential to limit larval dispersal across basins and support regional population subdivision, particularly between North Puget Sound (NPS) and Puget Sound Proper (PSP).

### 2.2 Washington Department of Fish and Wildlife recreational fishery survey 1977–2014 (Rec)

**Figure 4:**
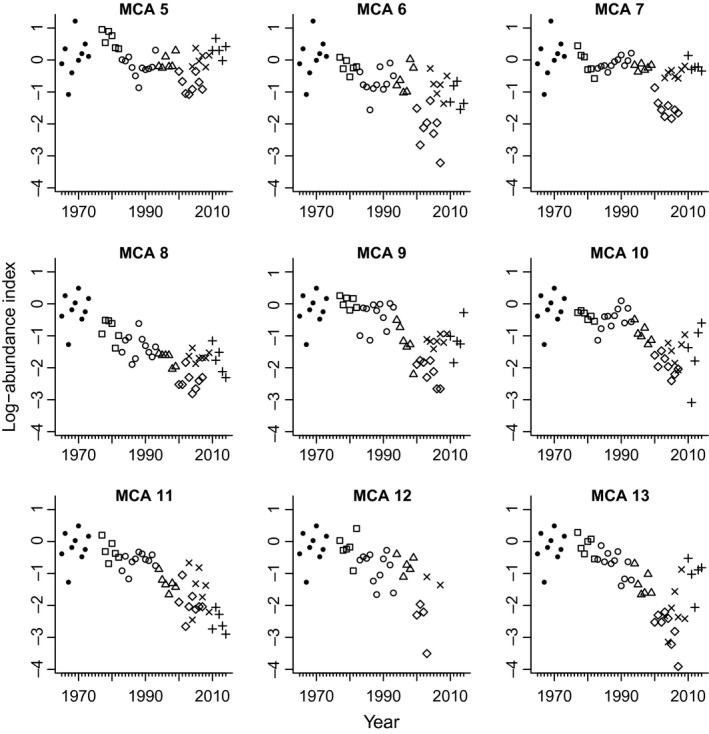
Log‐abundance index from the Washington Department of Fish and Wildlife (WDFW) recreational survey. The data are the log of catch (retained and released) per angler effort (catch‐per‐unit effort, (CPUE), where effort is defined as one angler trip. The different symbols represent periods with different bag limits. Note that the + and × are data obtained using a new methodology that includes a phone survey in addition to a creel (dockside) survey. The new methodology yields CPUE values that are higher than the prior method (see overlapping years with open diamonds and ×)

The Washington Department of Fish and Wildlife (WDFW) has surveyed the number of rockfish caught by recreational anglers (mainly boat based) in Puget Sound since the mid‐1960s using punch cards sent in from anglers, phone interviews, and creel (dockside) surveys (Figure 4). These surveys include identification of retained catches, although species identification for released fish has not been consistent over time. See the Supporting Information for details and references. We use the recreational CPUE, which quantitates total rockfish catch (both retained and released) per angler trip in nine MCAs (no. 5–13 in Figure 3) within Puget Sound. Following Drake et al. (2010), we define NPS as MCAs 5–7 and PSP as MCAs 8–13. We use greater Puget sound (GPS) to refer to MCAs 5–13. While some data exist for 1965–1973 (described in the Supporting Information), they are highly variable and use inconsistent geographic designations, and thus are not used in our analysis of growth rates. We do use these data for estimating changes in species composition (Figure 4).

In response to declines in abundance, the WDFW reduced the allowed daily bag limits successively from the early 1980s onward, resulting in six regulatory periods which we include in the MARSS analysis(Williams et al., 2010):

1. 1977–1982: no catch limit
2. 1983–1993: ten rockfish per day in NPS and five per day in PSP.
3. 1994–1999: five per day in NPS and three per day in PSP.
4. 2000–2009: one per day in GPS but zero retention of canary and yelloweye rockfishes after 2001. The analysis for the original ESA‐listing (Drake et al., 2010) used data through 2007. Thus, the actual time series for period (4) run from 2000 to 2007.
5. 2000—2009: As for (4) above. However, in 2012, WDFW updated their methodology for re‐estimated CPUE from 2003 onward (See Supporting Information). We include these updated estimates for 2003–2010. Thus, there is temporal overlap between (4) and (5). Note that in 2004 retention of all rockfish species was prohibited in MCA12 (Hood Canal), which is captured by (5) with a one‐year discrepancy.
6. 2010–2014: No allowed retained catches except in the western portion of MCA 5 where catching black rockfish *S. melanops* and blue rockfish *S. mystinus* was allowed (three‐fish bag limit of either species). Additionally, bottom fishing below 120 feet (~36 m) was prohibited in GPS (reducing fishable area by approximately 70%) to avoid incidental catch of the three listed rockfish species, which tend to inhabit deeper waters.

These regulatory changes led to a drop in the recreational CPUE reported from one regulatory period to the next. Note that the reported CPUE is not zero after 2010, because released fish are recorded in phone surveys and dockside creels, and illegal retention is reported in both. Analysis of the recreational survey data by standard, univariate PVA is not possible because there are multiple time series, one for each of the nine MCAs for six different regulatory periods. However, it is straightforward to analyze these data in a MARSS framework.

### 2.3 Reef Environmental Education Foundation scuba diver surveys 1998–2014

**Figure 5:**
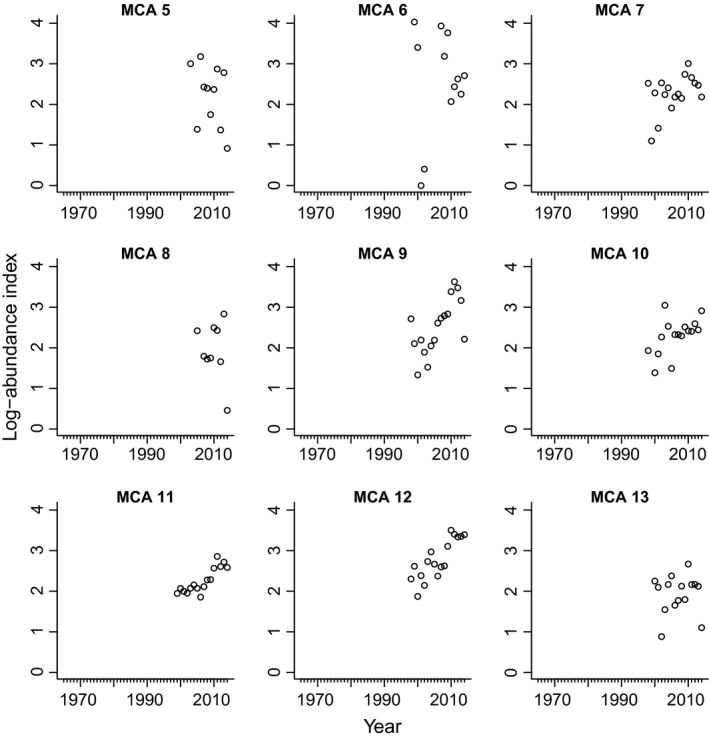
Reef Environmental Education Foundation (REEF) survey data used in the MARSS analysis by Washington marine catch areas (MCA). North Puget Sound (NPS) = MCAs 5–7; Puget Sound Proper (PSP) = MCAs 8–13. The data do not include young‐of‐year or Puget Sound Rockfish *S. emphaeus*

Reef Environmental Education Foundation (REEF) is a citizen science organization that trains recreational scuba divers to identify and record fish species during recreational dives (REEF, 2014). At the time of our analysis, Puget Sound data were available for years 1998–2014 (Figure 5). The data are reported in abundance categories: 1 fish, 2–10 fish, 11–100 fish, and ≥101 fish. Following Drake et al. (2010), we converted these abundance categories to minimum values (1, 2, 11, or 101 fish). We then averaged observations by site within years to control for more frequent surveys at popular dive sites and limited data for dives to hard‐bottom sites. Because we are averaging across a large number of sites, the REEF data used in our analysis are not categorical, but are instead averages with normal distributions. We calculated a yearly mean for each MCA, but excluded Puget Sound rockfish, *S. emphaeus*, from the analyses because they are much smaller than other rockfishes, can occur in a very high abundance ephemerally, and are not caught in recreational fishery. We also excluded young‐of‐year fish to eliminate recruitment pulses and to make the data more comparable with the recreational fishery and trawl survey data.

### 2.4 WDFW trawl survey 1987–2014 (Trawl)

**Figure 6:**
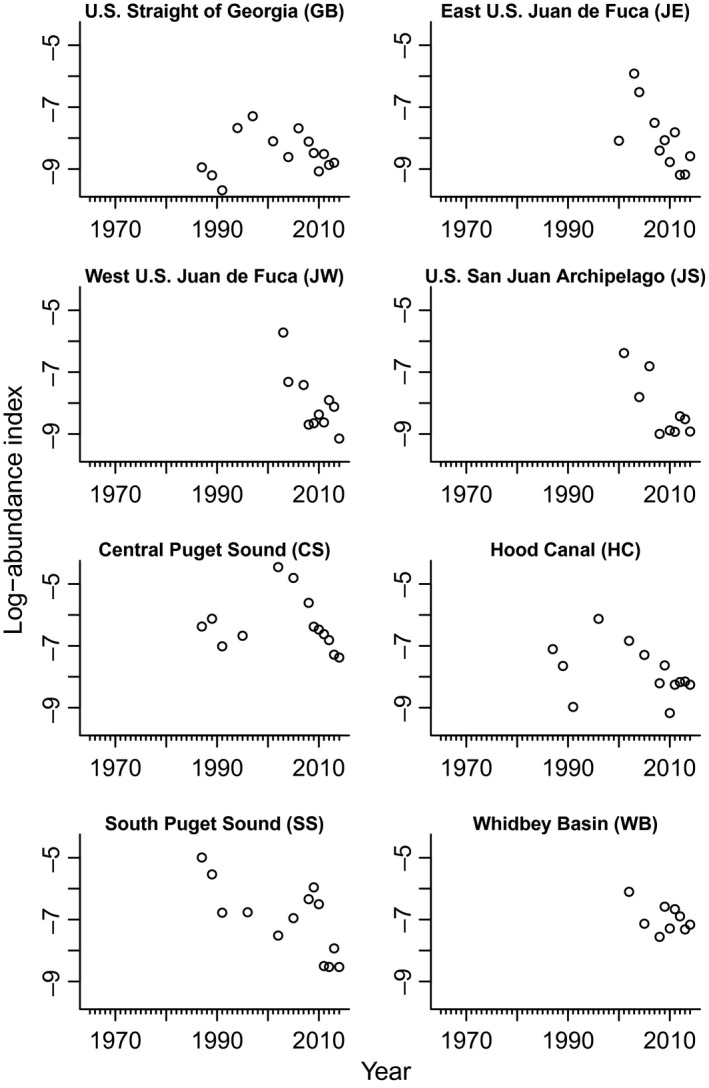
Washington Department of Fish and Wildlife (WDFW) trawl survey catch‐per‐unit effort [CPUE (number per km^2^)] by trawl area. GB = US Strait of Georgia, JE = east US Juan de Fuca, JW = west US Juan de Fuca, JS = San Juan Islands, HC = Hood Canal, CS = central Puget Sound, SS = South Puget Sound, WI = Whidbey Island Basin. GB, JE, JW, and JS comprise North Puget Sound (NPS). CS, HC, WI, and SS comprise Puget Sound Proper (PSP)

Since 1987, the WDFW has conducted a depth‐stratified fishery‐independent trawl survey throughout Puget Sound. Effort is allocated among 12 regions throughout Puget Sound, nine of which are in US waters. Here, we use data for eight of the nine US regions (Figure 6), excluding one (Discovery Bay) because it had only two data points across all years. CPUE (number per m^2^) for total rockfishes was calculated by dividing the total number of individuals (all rockfish species) caught by the swept area of the trawl. We then calculated the mean CPUE for each year for each of the eight trawl regions. Although the survey design shifted after 2008 from random allocation of sampling effort to index sites, we do not model these as separate time series because the general habitat (soft bottom), depth range, and overall sampling methodology were similar before and after the shift (See the Supporting Information for further details). The Trawl survey samples mainly soft bottom habitat and may sample a different rockfish assemblage than that sampled by the Rec and REEF surveys, which sample rocky habitats. The listed rockfish species are found mostly in rocky habitats and are represented infrequently in the Trawl survey.

### 2.5 Time‐series analysis

We used MARSS models (Hinrichsen & Holmes, 2009; Holmes et al., 2012, 2014) to estimate two key PVA parameters for “total rockfish” (summed abundance across rockfish species) in Puget Sound: long‐term population growth rate and process variance. Although total rockfish is a multi‐species assemblage, rather than a single species population, the autoregressive process model can approximate sums of population trajectories (Holmes & Semmens, 2004). Our process equation, which models a set of true, but unknown, abundance trajectories on a log‐scale, takes the form:

(1a) $$\left[\begin{array}{c} x_{1,t}\\ x_{2,t}\\ \vdots \\ x_{m,t} \end{array}\right]=\left[\begin{array}{c} x_{1,t-1}\\ x_{2,t-1}\\ \vdots \\ x_{m,t-1} \end{array}\right]+\left[\begin{array}{c} w_{1,t}\\ w_{2,t}\\ \vdots \\ w_{m,t} \end{array}\right],\;\text{where}\left[\begin{array}{c} w_{1,t}\\ w_{2,t}\\ \vdots \\ w_{m,t} \end{array}\right]∼\text{MVN}\left(\left[\begin{array}{c} u_{1}\\ u_{2}\\ \vdots \\ u_{m} \end{array}\right],Q\right)$$

where *x*_*i,t*_ is the state, in this case (log) abundances of rockfish in year *t* for the *i*th abundance trajectory. This equation replicates the standard population model equation underpinning a count‐based PVA, but in multivariate form. Each *x*_*i*_ is a population trajectory (Figure 1b), which might be independent or co‐vary temporally. The latter means that good versus bad years may correlate to some degree, even if their long‐term population growths (*u*_*i*_) are quite different. The *w*_*i,t*_ variable signifies process errors, which represent the population growth rate in year *t*. The process errors are modeled as multivariate normal (MVN) with variance‐covariance matrix ***Q***. The mean of *w*_*i,t*_ is *u*_*i*_ *,* the average population growth rate for population *i*. For example, for a model with distinct north and south rockfish populations sharing a common average growth rate *u*, the same level of process variance (*q)* and correlated yearly growth rates (*c*) would be modeled as

$$\left[\begin{array}{c} x_{N,t}\\ x_{S,t} \end{array}\right]=\left[\begin{array}{c} x_{N,t-1}\\ x_{S,t-1} \end{array}\right]+\left[\begin{array}{c} w_{N,t}\\ w_{S,t} \end{array}\right]\;\text{where}\left[\begin{array}{c} w_{N,t}\\ w_{S,t} \end{array}\right]∼\text{MVN}\left(\left[\begin{array}{c} u\\ u \end{array}\right],\left[\begin{array}{cc} q & c\\ c & q \end{array}\right]\right).$$

The second part of our model is the observation equation, which relates the observed data to the abundance trajectories (the *x* in Equation 1a). It takes the form

(1b) $$\left[\begin{array}{c} y_{1,t}\\ y_{2,t}\\ \vdots \\ y_{n,t} \end{array}\right]=Z\left[\begin{array}{c} x_{1,t}\\ x_{2,t}\\ \vdots \\ x_{m,t} \end{array}\right]+\left[\begin{array}{c} a_{1}\\ a_{2}\\ \vdots \\ a_{n} \end{array}\right]+\left[\begin{array}{c} v_{1,t}\\ v_{2,t}\\ \vdots \\ v_{n,t} \end{array}\right]$$

where the *v*_i_ variables are the process errors and are MVN with a mean of zero. *y*_*j,t*_ is the number of observations (possibly with missing observations) in year *t* for the *j*th observation time series. ***Z*** is a [0,1] matrix that defines how the observations relate to the underlying abundance trajectories. For example, suppose we have observation time series in four locations: three sampling the northern population and one sampling the southern population. We can model this in a MARSS framework as

$$\begin{array}{cc} \left[\begin{array}{c} y_{1,t}\\ y_{2,t}\\ y_{3,t}\\ y_{4,t} \end{array}\right]= & \left[\begin{array}{cc} 1 & 0\\ 1 & 0\\ 1 & 0\\ 0 & 1 \end{array}\right]\left[\begin{array}{c} x_{N,t}\\ x_{S,t} \end{array}\right]+\left[\begin{array}{c} 0\\ a_{2}\\ a_{3}\\ 0 \end{array}\right]+\left[\begin{array}{c} v_{1,t}\\ v_{2,t}\\ v_{3,t}\\ v_{4,t} \end{array}\right]\;\\ & \text{where}\;\left[\begin{array}{c} v_{1,t}\\ v_{2,t}\\ v_{3,t}\\ v_{4,t} \end{array}\right]∼\text{MVN}\left(0,\left[\begin{array}{cccc} r & 0 & 0 & 0\\ 0 & r & 0 & 0\\ 0 & 0 & r & 0\\ 0 & 0 & 0 & r \end{array}\right]\right) \end{array}$$

The *a* variables are scaling terms for each observational time series, which allows time series estimated across different scales to be combined (Figure 1a). For our study, we model the successive reductions in angler CPUE in the Rec survey caused by reductions in bag limits as separate time series observing the same trajectory. Because the absolute scaling of the abundance trajectories is unknown, the estimated population trajectories are indices; they relate to the true abundances by an unknown scaling factor. A mathematically equivalent (but computationally slower) way to model the bag‐limit changes would be to use time‐varying *a*, such that there is one *y* for each Rec (in each MCA) and a different *a* for each bag‐limit period. We were not willing to assume that the effect of the bag‐limit on fishing behavior would be the same across MCAs. Thus, the *a* variables also account for different CPUE values in different MCAs when the MCAs are combined into regional trajectories (NPS and PSP) or a single GPS trajectory.

Reducing the bag limit caused a step reduction in CPUE because fishers’ daily catches were restricted. Implicit in the model is the assumption that whatever caused step changes in the Rec survey (observed CPUE) did not also cause step changes in the true population abundance. There is no reason to assume that a bag‐limit change would cause an immediate, step change in the actual abundance of rockfish given the long life‐span of rockfishes and the relatively long period needed to reach sexual maturity for many species (Love et al., 2002). The intent of reduced bag limits was indeed to increase population size, but any resultant increase would be expected to be gradual. Although the Trawl survey is fishery‐independent and free from effects of changes in fisher behavior, it came into full use after 2000. Thus, it does not support studies of long‐term abundance changes. Additionally, few of the listed species are captured in the Trawl survey because it does not sample rocky habitats. The species found in rocky habitats are caught in the Rec survey. It should also be noted that although the Trawl survey is fishery independent, it is not free of survey effects. The Trawl survey uses index sites and thus may be prone to localized survey‐induced depletion. The Rec survey, though fishery dependent, surveys a wide variety of habitats and is spatially extensive.

### 2.6 Tests of estimation performance

Figure 1a illustrates the relationship between the underlying (and hidden) population trajectory represented by *x* in Equation 1a and the observations represented by *y* in Equation 1b. Note that the population trajectory is not a straight line, but rather fluctuates year‐to‐year with local periods of increases and decreases. Here, population abundance is modeled as a stochastic auto‐regressive process, the same model used in a classic count‐based PVA model. In Figure 1a, the observation time series are shown as a series of non‐overlapping segments with different scaling factors; the Rec data take this structure because of the consecutive changes in the bag limits. The scaling factors can be estimated for non‐overlapping segments because we assume that they all follow the same underlying population trajectory (black line in Figure 1a). Estimation works best when the segments are long, separated by no or only small gaps. Replication (i.e., multiple time series following the same population trajectory at the same time) improves estimation of the scaling factors greatly.

We used simulation studies to test whether our models were able to return unbiased estimates of the key parameters accurately using simulated data with the same structure (number of time series, gaps, lengths, and levels of variance) as our data. Figure 2 shows simulation results obtained with data similar in structure to the NPS Rec survey. The data consist of five non‐overlapping segments (6, 11, 6, 10, and 5 years long) with three spatial replicates (MCAs) (Figure 2a). This structure is similar to that of our NPS Rec data. The simulated data appear to have a strong upward trend due to progressively increasing scaling factors for successive segments (done by design to create this illusion). However, the true population trend is negative (3.8% decline per year). The points in Figure 2b show an example of the true population trends in one simulation. Figure 2c shows that the model estimates the declining trend correctly even though the data appear to have an increasing trend (Figure 2a). If the process variance estimate is non‐zero, it is also unbiased (Figure 2d). For this simulation, the process variance estimates were frequently zero. In practice, if a non‐zero variance estimate occurs, one can examine the likelihood surface to find the interior local maximum where all the variances are non‐zero. In our case study, the variance estimates were always non‐zero except when the model conflicted observably with the covariance structure in the data. There are limitations to how much data fragmentation can be handled, which depend on the lengths of the segments and the amount of spatial replication in the data. The Supporting Information includes the R code used for the simulation shown in Figure 2. This code can be adapted for testing performance with other data structures.

### 2.7 Analysis of spatial and survey structure in the data

Our primary goal was to estimate the average long‐term population growth rate and the process variance for total rockfish abundance in Puget Sound over our study period. We used model selection to test the data support for different spatial structures (specifically, temporally independent subgroups) within the rockfish assemblage in Puget Sound because model structure can affect estimates of population growth rate. The model selection analysis was repeated using three different data combinations: (1) Rec only; (2) Rec and REEF; and (3) Rec, REEF, and Trawl. We ran the analyses with these different datasets to determine whether and how the results and associated conclusions were influenced by dataset characteristics. Providing a clear demonstration of how particular datasets affect the results for stakeholders is important because the conclusions developed based on the results have direct management implications, which in turn affect resource users throughout the Puget Sound region.

We tested support for three spatial structures by manipulating the ***Z*** matrix in Equation 1b in the following three ways:

1. fine‐scale structure (one rockfish trajectory for each MCA);
2. regional structure (rockfish trajectories for NPS and PSP; and
3. no spatial structure (a single rockfish trajectory for all of GPS).

These three spatial structures reflect the hydrographic basins and habitat structure (prevalence of rocky habitat) that have the potential to create temporally independent local populations of rockfish. For analyses involving more than one data source (b and c above), we also allowed that each survey could be sampling congruous or distinct rockfish assemblages. Thus, for example, to treat the Rec and Trawl surveys as sampling distinct GPS‐wide assemblages, a model with two rows in the *x* matrix in Equation 1a (one for Rec_GPS_ and another for Trawl_GPS_; See Tables 1, 2, and S1 for model sets). The Rec data appear in the observation model, Equation 1b, as independent time series of total rockfish for each of the nine MCAs in each of the six regulatory periods (*n *=* *54). There were nine REEF time series (one for each MCA) and eight Trawl time series (one for each WDFW trawl area). Thus, for the analysis using all three data sources, the *y* matrix in Equation 1b has 71 rows.

**Table 1:** Multivariate autoregressive state‐space (MARSS) models tested using the WDFW recreational survey data only. For each ***Z*** structure, all combination of the *u* and ***Q*** structures were tested. In columns 1 and 2, Region = 2 trajectories or growth rates for NPS and PSP, MCA = trajectories or growth rates for each of the 9 MCAs, and GPS = one Greater Puget Sound trajectory or growth rate. Cov = covariance between all population trajectories, No cov = no covariance among trajectories. Separate process variance was estimated for each trajectory. There were 54 observation time series. The number of estimated parameters for each term is in parentheses. For column 1, this is the number of initial states (**x**_0_) and number of *a* in **a**. There were nine **R** parameters for each model

***Z*** structure: Number and structure of trajectories (**x**_0,_ **a**)	Growth rates (**u**)	Covariance structures (***Q***)
MCA (9, 45)	MCA (9)	Cov (45)
	Region (2)	No cov. (9)
	GPS (1)	
Region (2, 52)	Region (2)	Cov (3)
	GPS (1)	No cov. (2)
GPS (1, 53)	GPS (1)	No cov (only one trajectory) (1)

For each spatial structure, we tested shared or unique average population growth rates *u*. Two populations could have different population trajectories but share the same long‐term population growth rate (example in Figure 1b). If the data support a common population growth rate across regions, then we can use data from both regions to estimate the *u* parameter. Because *u* is a key parameter, we aim to maximize the amount data used to estimate it. We allowed the population growth rates to differ across all population trajectories in the model (by region/survey type) or forced it to be equal for all. For example, the model might specify that each MCA samples an independent trajectory but that those trajectories each have the same average growth rate. We also tested shared or unique process variance across trajectories. Via the ***Q*** covariance matrix, we allowed the trajectories to be either temporally correlated (i.e., good and bad years are correlated) or independent. We tested full covariance (all trajectories allowed to covary), covariance only between regions (NPS and PSP), or covariance only within surveys (for models where surveys were given different trajectories). For all models, we assumed independent obs. Because the data were log‐transformed, the proportional observation errors (10% up and 10% down) were modeled as normally distributed.

The data supporting each model were evaluated with AICc (Akiake's Information Criterion corrected for sample size) and the models were ranked by ΔAICc (the AICc minus the AICc for the model with lowest AICc)(Burnham & Anderson, 1998). Models with ΔAICc < 2 were considered to be similarly supported. Although we conducted three model selection analyses using three different datasets (Rec only; Rec plus REEF; or Rec, REEF plus Trawl), for any given model selection analysis, AICc values were only compared within model sets fit to the same data. Our models were all nested models of the most complex (full) model, which is important when using AICc and a benefit of using a MARSS framework. Confidence intervals and standard errors were computed using the Hessian approximation for the best‐supported models. Analyses were run in R 3.1.1 (R Core Team 2014) with the MARSS package v.3.9 (Holmes et al., 2012).

### 2.8 Relative trends for the ESA‐listed species

Because the three ESA‐listed species are rarely caught, we had no yearly data with which to analyze their population trajectories of the species directly. However, we did have information on decadal changes in the species composition of recreational catches (specifically the proportions of the listed species), which we used infer population growth rates for these three species relative to changes in total rockfish abundance—essentially setting an upper or lower bound. For example, if the frequency of a listed species increased over time, we would infer that the species increased more quickly (or decreased more slowly) than did the total rockfish abundance; if its frequency decreased, we would infer it had increased more slowly (or decreased more quickly).

For the years from 1965 to 2007, we took species composition information directly from the study by Drake et al. (2010; Tables 11–18). For 2008–2014, we used raw data from the WDFW creel survey. Note, the extreme reductions in bag limits from 2000 onward, prohibition on retention of canary and yelloweye in 2001, and inability to fish in deeper waters that commenced in 2010 may have reduced observations of the listed species in the assemblage data for the last two decades (2000 and 2010) because fishers were likely to adjust their activities to avoid the listed species. We computed decadal averages by summing the counts by decade and then converting decadal counts of the three listed species to proportions of the total assemblage. We used the sampling effort to estimate 95% confidence intervals for species proportions using Wilson intervals (Agresti & Coull, 1998). No information on sampling effort was available for the 1960s and 1970s, however. We did not use information from the REEF and Trawl surveys, because they cover a shorter time period and the listed species were rare in those samples. See the Supporting Information for the data and references.

## 3 Results

### 3.1 Spatial structure in the rockfish assemblage

**Figure 7:**
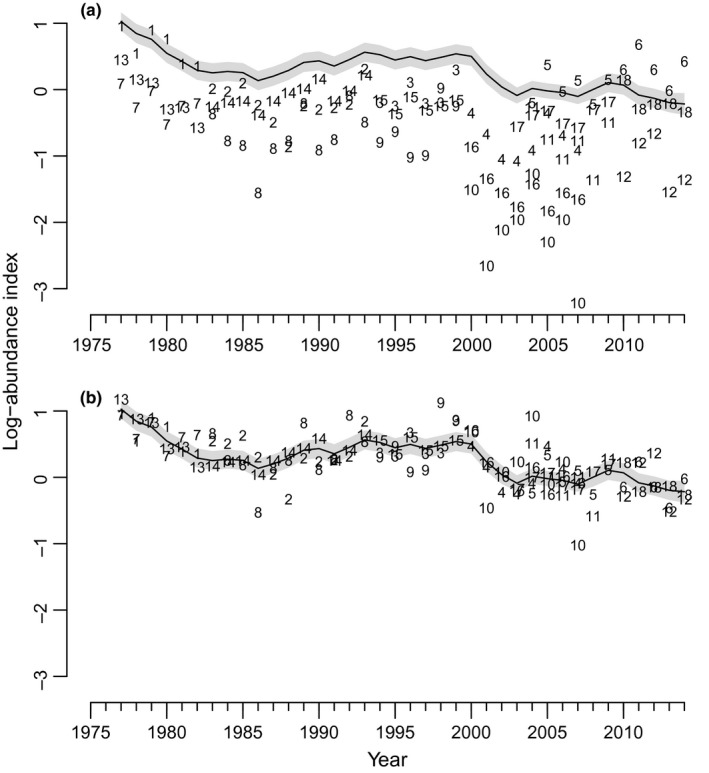
Estimated trajectory (solid line) for total rockfish in North Puget Sound [NPS (Rec_NPS_)] from the best‐supported model using the Rec data only showing the effect of the scaling parameter *a*. Numbers refer to separate Rec time‐series for each regulatory period in marine catch areas (MCAs) 5–7. (a) Raw data for each NPS time‐series, and (b) data for each NPS time‐series corrected by the scaling parameter *a*. Grey envelopes indicate 95% confidence intervals

**Figure 8:**
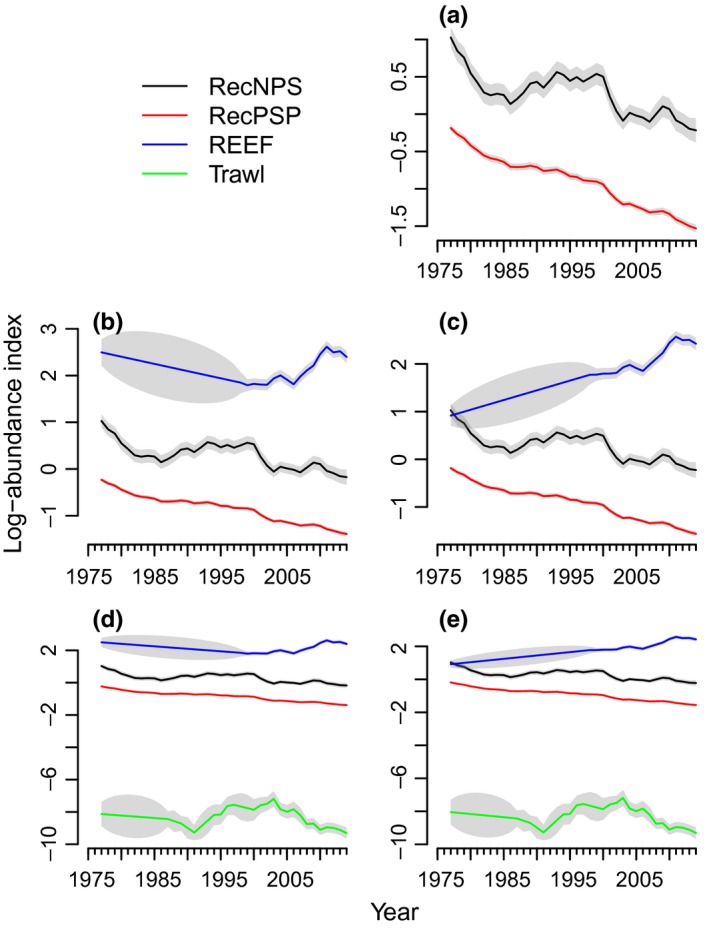
Estimated trajectories for total rockfish based on the best‐supported models. (a) Recreational Fishery Survey (Rec) data only: one *u,* two states, *u*_*GPS*_ = −0.038. (b) Rec + REEF data: one *u*, three states, *u*_GPS_ = −0.031. (c) Rec + REEF data: two *u’*s, three states, *u*_Rec_ = −0.039, *u*_REEF_ = 0.041. (d) Rec + REEF + Trawl data: two *u*'s, four states, *u*_Rec/Trawl_ = −0.039, *u*_REEF_ = 0.041. (e) Rec + REEF + Trawl data: one *u*, four states, *u*_GPS_ = −0.032. Grey envelopes indicate 95% confidence intervals

The model that best fit the Rec data alone had separate (but temporally correlated) trajectories for total rockfish (sum of all species) in NPS and PSP (denoted as Rec_NPS_ and Rec_PSP_; Tables S2 and S3; Figures 7 and 8a). There was little support for fine‐scale spatial structure (independent trajectories at the MCA level) or no spatial structure [single trajectory for GPS (Table S2)]. When both the Rec and REEF survey data were used, two models had similar levels of support with only a 1.4 difference in AICc (Table S4‐S6). Both models included three trajectories: two regional Rec trajectories (Rec_NPS_ and Rec_PSP_), plus a separate REEF trajectory (Tables S5 & S6). These most robustly supported models indicated correlation between the Rec trajectories but not between these and the REEF trajectory. The estimated trajectories for these models are shown in Figure 8b,c. The results obtained based on all three datasets were similar to the aforementioned results (Table S7‐S9, Figure 8d,e). Four models had ΔAICc values less than 2.0. All had two regional Rec trajectories (Rec_NPS_ and Rec_PSP_) and a separate REEF trajectory for all GPS. The top three models had a single all‐GPS trawl trajectory, whereas the 4th model had two regional Trawl trajectories: Trawl_NPS_ and Trawl_PSP_.

Thus overall, the models that received the strongest data support treated each survey as sampling a separate rockfish trajectory. There was little data support for fine‐scale spatial structure but strong support for regional (NPS vs. PSP) structure in the rockfish assemblage surveyed by the recreational fishery. There was some support for NPS versus PSP structure in the assemblage sampled by the trawl survey but none for that sampled by the REEF survey. However, these two surveys are shorter time series, and the lack of data support may be a reflection of the data rather than the underlying assemblages tracked by these surveys.

### 3.2 Long‐term growth rate and process variance

None of the top models included spatial structure in *u* (meaning a different population growth rate in different MCAs or in NPS vs. PSP); see Tables S2, S4, and S7 for the models sorted by data support (ΔAICc). In addition to no spatial differences in *u*, the top models also did not support different *u* the Rec and Trawl surveys. These findings suggest that the rockfish assemblages sampled by the Rec and Trawl surveys have experienced similar rates of population growth across all of Puget Sound. Conversely, the best models did support a different *u* for the REEF survey, suggesting that this survey, which targets more shallow waters, may track a different assemblage.

To summarize, the top model using all three datasets had four trajectories (Tables S7 and S10). The Rec_NPS_, Rec_PSP_, and Trawl trajectories shared a common negative *u* (*u = *−0.039 ± 0.01), indicating an average of 3.9% per annum decline (a 77% decline from 1977 to 2014). The REEF trajectory had a positive *u* (*u = *0.041 ± 0.038), indicating an average of 4.1% per annum increase since 1998. This result mirrored the results obtained for the Rec and REEF data without inclusion of the Trawl data (Table S4). The 2nd and 3rd best models encompassing all three datasets had a single shared *u* across all trajectories (*u = *−0.032 ± 0.011; Table 2, last row) but differed in covariance structure (Table S8). These models had one fewer parameters, but this simplification came at the cost of an increased process variance for the REEF trajectory in that the single declining *u* estimated by this model conflicted with the increasing trend in the REEF data (Figures 5 and 8). In general, the process variance matrix could be estimated for all models, except those models that did not allow covariance between the Rec_NPS_ and Rec_PSP_ trajectories. The process variance estimates were in the range of 0.03 to 0.01, which is similar to ranges found for marine fish populations (Holmes et al., 2007). The observational variance differed among MCAs by as much as an order of magnitude and was generally one to two orders of magnitude greater than the process variance (Tables S3, S5, and S8). The different MCAs are characterized by differing effort levels (the number of angler trips) and thus different observation variances among them is expected.

**Table 2:** Models tested using the WDFW recreational survey and REEF scuba survey data. For each ***Z*** structure, all combination of the *u*'s and all ***Q*** structures were tested. The geographic designations are North Puget Sound (NPS), Puget Sound Proper (PSP), MCA = management conservation area, and GPS = Greater Puget Sound. All models included separate process variance for each rockfish trajectory with either no covariance between the trajectories or allowing covariance between all or some of the trajectories; see process covariance column with additional information in the footnotes. There were 63 observation time series: 54 Rec (nine MCAs and six regulatory time periods) and nine REEF (one for each MCA). Numbers in parenthesis are the number of estimated parameters for term. For the population trajectories, the estimated parameters are the initial value of the trajectory (**x**_0_) and the scaling parameters (*a*). The number of *a* in **a** was 54 (the number of observation time series) minus the number of trajectories. Separate observation variance was estimated for each survey in each MCA

***Z*** structure: Number and structure of trajectories (**x**_0,_ **a**)	Growth rates (**u**)	Covariance structures (***Q***)
MCA × Survey (18, 45)	MCA × Survey (18)	All trajectories covary (171)
	Region × Survey (4)	No covariance (18)
	Region (2)	
	Survey (2)	
	GPS (1)	
Region × Survey (4, 59)	Region × Survey (4)	All trajectories covary (10)
	Region (2)	Regions covary within surveys (6)
	Survey (2)	Surveys covary within regions (6)
	GPS (1)	No covariance (4)
Rec_NPS_, Rec_PSP_, REEF (3, 60)	Rec_NPS_, Rec_PSP_, REEF (3)	All trajectories covary (6)
	Survey (2)	Rec_NPS_ and Rec_PSP_ covary (4)
	GPS (1)	No covariance (3)
Rec, REEF_NPS_, REEF_PSP_ (3, 60)	Rec, REEF_NPS_, REEF_PSP_ (3)	All trajectories covary (6)
	Survey (2)	REEF_NPS_ and REEF_PSP_ covary (4)
	GPS (1)	No covariance (3)
Survey (2, 61)	Survey (2)	All trajectories covary (3)
	GPS (1)	No covariance (2)
Region (2, 61)	Region (2)	All trajectories covary (10)
	GPS (1)	No covariance (2)
GPS (1, 62)	GPS (1)	No covariance (2)

Overall, allowing spatial and survey structure in the model improved the fit of the model (as measured by log likelihood). These allowances also affected the estimates of the long‐term growth rate of the Puget Sound rockfish assemblage. Treating the REEF surveys as having sampled the same assemblage as the Rec and Trawl data led to a lower estimated rate of year‐to‐year decline (3.2% vs. 3.9%). Figure S1 summarizes how the long‐term trend (*u*) estimates depended on the model structure. Models with a structure (overly complex or overly simple) that was poorly supported by the data tended to estimate less severe decline than the best‐supported models (estimates closer to 0 in Figure S1). This tendency illustrates that model structure, specifically with respect to one's assumptions about how the data are related to the population one is studying, affects the long‐term trend estimates.

### 3.3 Species composition

**Figure 9:**
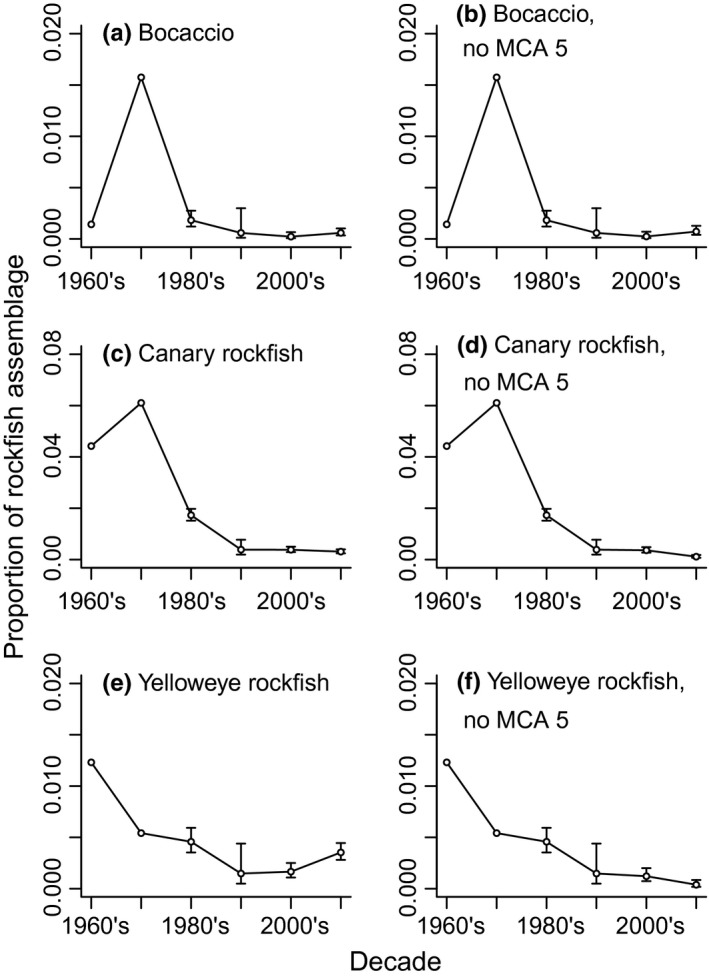
Prevalence of bocaccio, canary, and yelloweye rockfishes as a proportion of the total rockfish assemblage in the Washington Department of Fish and Wildlife (WDFW) recreational Survey for marine catch areas (MCAs) 5‐13 or 6‐13. a) bocaccio MCAs 5‐13, b) bocaccio MCAs 6‐13, c) canary rockfish MCAs 5‐15, d) canary rockfish MCAs 6‐13, e) yelloweye rockfish MCAs 5‐13, f) yelloweye rockfish MCAs 6‐13. MCA 5 is closest to the coast in the Strait of Juan de Fuca and is not included in the population designation for the listed species. Error bars indicate 95% confidence limits. Data are shown in Table S11.

All the three ESA‐listed species declined as a proportion of the rockfish caught (released + retained) in the recreational fishery in Puget Sound (Figure 9; Table S11), suggesting that they have declined to a similar or greater extent than did the abundance of total rockfish (sum of all species). The peak catch of bocaccio was in 1970 (approximately 2% of rockfish caught by recreational anglers), but has since declined and remained low or zero (Figure 9a). Canary rockfish made up 2–6% of the recreational catch in the 1960s (Figure 9c), then peaked in relative abundance in the 1970s; its abundance has since declined and remained low. Yelloweye rockfish showed a similar pattern to canary, increasing in prevalence over time in the limited data available for the 1960s, reaching 3–4% of the catch in the 1970s (Figure 9d); its abundance then declined to less than 0.5% of the recreational catch in the 2000s. Canary and yelloweye also decreased as a proportion of the sightings in the REEF surveys from the mid‐2000s onward. However, species assemblage data for the Rec survey in the 2000s and 2010s should be taken with some caution due to the potential shifts in fishing effort mentioned earlier (Figure S2). Reductions in total catch would also reduce the probability of catching one of the listed species. Nevertheless, we would still expect to see more individuals of the listed species than observed here if their relative abundances had remained constant (Drake et al., 2010). There were small increases in the relative abundances of canary and yelloweye rockfishes in recreational catches in the 2010s owing to increased catch levels in MCA 5, which is considered part of the coastal population. When we exclude MCA 5, the frequency of canary and yelloweye rockfishes in the total catch declines (Figure 9d,f). The earlier declines in the Rec survey data, recent declines in the REEF survey data, and equivocal evidence from recent recreational data from the 2000s onward suggest that, based on the best‐available evidence and a precautionary approach, we should assume that these species either followed the trend for the Rec and Trawl trajectories (3.8–3.9% average yearly declines) or, potentially, decreased at a greater rate.

## 4 Discussion

MARSS models allow key parameters for PVA, namely long‐term population growth rate and process variance, to be estimated in situations where constraints and irregularities in the available data limit the use traditional univariate methods used in count‐based PVA. Limiting constraints and irregularities include multiple types of surveys, surveys conducted in different regions, missing data, and temporal changes within surveys. In this study, we used MARSS models to combine data from three different rockfish surveys to estimate the long‐term growth rate of and process variance parameters for a multi‐species rockfish assemblage in Puget Sound, WA. Each survey had regional structure, and one (Rec) was strongly affected by changes in management over time. MARSS models allowed us to analyze all of these data in a single, unified multivariate time‐series framework and to provide metrics for parameter uncertainty and model selection. Importantly, it allowed us to include spatial replication as well, which is critical for separation of process and observation variance parameters.

One of the strengths of MARSS modeling is that it allows one to model structure in survey data and in underlying population (in our case, abundance of total rockfish) dynamics. Estimating this structure is important because it influences estimates of long‐term growth rate and process variance (Figure S1). In addition, the underlying spatial structure in a population's dynamics has many implications for wildlife management in terms of spatial management plans and portfolio effects (Hilborn et al., 2003). We found strong empirical support for four rockfish trajectories: two regional trajectories (NPS and PSP) tracked by the Rec data and two GPS‐level trajectories (REEF_GPS_ and Trawl_GPS_) tracked by the REEF and Trawl data, respectively. Furthermore, there was support for both medium‐scale spatial structure and survey structure. There are clear reasons for the latter. Note, the surveys sample different depths and habitat types, and thus might be expected to sample different rockfish assemblages. There are several potential reasons for the spatial structure. Rocky habitat is more common in the northern part of Puget Sound (Williams et al., 2010), and the species composition within the recreational catch differed between NPS and PSP, with there being substantially more black rockfish and to a lesser extent yellowtail rockfish *S. flavidus*—both of which are semi‐pelagic, schooling species (Love et al., 2002)—in NPS catches than in PSP catches. The lack of spatial structure in the REEF and Trawl surveys could be due to insufficient data given that these surveys have many fewer data points (shorter time series) than the Rec survey. This caveat likely applies to the Trawl survey data because the models with NPS and PSP Trawl trajectories did fall just within the ΔAICc < 2 threshold of the top model.

MARSS allowed us to combine time series from different surveys and regions when estimating a population trend. However, when the data support separate growth rates for different surveys, one must use biological knowledge to choose the most relevant estimate. The best‐supported model using all three surveys gave two long‐term growth rate estimates: 3.8–3.9% per year decline for the rockfish assemblages sampled by the Rec and Trawl data, and 4.1% per year increase for the rockfish assemblage sampled by the shallower REEF surveys. Because all three datasets reflect changes in adult numbers, the increases observed in the REEF survey cannot be attributed to pulses of juvenile recruits. The Rec survey (3.8–3.9% decline) is likely to be the most relevant for the purpose of inferring historical trends in the listed species. The REEF data are limited to a 40‐m (~130‐ft.) bottom depth (set by recreational diving limits; most dives were <20 m), and the three listed species are all more common below 50 m (Love et al., 2002). Thus, the REEF surveys may not sample an assemblage relevant to inferring changes in the broader abundance of the listed species, but they do suggest that shallow‐water rockfishes have shown strong recovery in Puget Sound since 1998. Both the Rec (prior to 2010) and Trawl surveys sample deeper depths, but the listed species, which are generally found on hard bottoms (Love et al., 2002), were only rarely encountered by the trawl survey. Nevertheless, model selection supported a shared rate of population decline for the rockfish assemblages sampled by the Rec and Trawl surveys.

While we cannot estimate population parameters for each of the ESA‐listed species, the analyses here can set some bounds. Although recent assemblage structure data should be interpreted with some caution due to potential changes in fisher behavior, as noted above, the relative abundance of each of these three ESA‐listed species decreased in the Rec survey, which suggests that they have declined at a the same or greater rate than the 3.8–3.9% per year estimated from the Rec and Trawl surveys. There have been recent increases in yelloweye, but that trend is restricted to MCA 5 in the Strait of Juan de Fuca, which is closest to the coastal population and was excluded from the listed species’ Distinct Population Segment (Drake et al., 2010). It should be noted that since 2010, recreational fishing for bottom fish has been restricted to depths less than 120 feet (36.6 m) to reduce impacts on canary and yelloweye rockfishes. This depth change and the restrictions on bottom‐fishing following the 2010 listing may lead to a qualitative change in the survey outcomes, perhaps causing them to align better with shallower REEF survey outcomes. Thus, caution is warranted in using future, shallower, and recreational rockfish catches as a trend indicator for the listed species.

The choice to model multiple survey time series as observations of the same underlying population trajectory requires forethought especially when the time series do not overlap temporally. If the surveys sample similar habitat and areas and are separated by only short temporal gaps, then they are likely to track the same underlying population. For example, we treated each regulatory period within an MCA as containing observations of the same population, albeit sampled in different years. The Rec data share methodology (angling) and general target community (bottom fish) across regulatory periods, although the 120‐ft depth limit imposed in 2010 has restricted the depth range of the sampled assemblage relative to earlier periods. The step reduction in CPUE from one regulatory period to the next due to a bag limit change can be modeled by the scaling parameter *a*. Although it is technically possible to set the scaling parameter a priori*,* we chose to allow the model to estimate *a* because we do not know exactly how fisher behavior changed with the reductions in catch limits. In 2008, the Trawl survey shifted from random samples to index sites. However, we did not model these as separate time series because the Trawl survey continued to target primarily soft‐bottom habitats. Future analyses might further investigate this assumption and the effect of shifting from random to index sampling.

In cases where there is time‐series overlap, such as with the Rec, REEF, and Trawl surveys, one can rely to a greater extent on model comparison to investigate combining or separating time series based on similarity in population growth rate, process, and observational variance. The data supported models with four separate rockfish trajectories: one for each survey, but with two regions for the Rec survey. These four trajectories shared two population growth rates: one negative population growth rate for the Rec and Trawl trajectories, but a separate positive growth rate for the REEF trajectory. In cases with little temporal overlap and substantial differences in survey methodology, specifics of the surveys and the biology of the populations being surveyed will be needed to guide decisions about whether to treat the surveys as samples of the same population, whether at the same point in time or at different points in time.

Providing scientifically sound estimates of the population trajectories and population growth rate parameters for species of conservation concern is essential not only for the conservation and management of those specific species, but also for management of other species due to efforts to reduce bycatch. These management decisions can have wide‐ranging impacts on fishing. For example, within Puget Sound, the listing of bocaccio, canary, and yelloweye rockfishes resulted in the development of maximum depth regulations for recreational bottom‐fishing to minimize associated bycatch, which in turn impacted fishing for lingcod *Ophiodon elongatus* and Pacific halibut *Hippoglossus stenolepis* (Anderson et al., 2013). Unfortunately, in many cases, inconsistencies among data sets make traditional methods for estimating population trajectories and growth rates difficult, or even impossible, to use. MARSS models provide a rigorous statistical framework for solving some of these challenges and can reduce the proportion of cases that are assigned a designation of “data deficient.”

## Conflict of Interest

None declared.

## Author Contributions

N. Tolimieri developed the study design, ran all MARSS analyses, and wrote the manuscript. E.E. Holmes developed MARSS models, developed the study design and simulation modeling, and wrote the manuscript. G. Williams contributed to writing the manuscript. R. Pacunski conducted fieldwork to collect the data and provided insights into dataset strengths and weakness. D. Lowry conducted fieldwork and assisted in writing the manuscript.
