# Supplementary material for: Population assessment using multivariate time‐series analysis: A case study of rockfishes in Puget Sound
Source: Ecol Evol. 2017 Mar 21;7(8):2846–60. doi: 10.1002/ece3.2901 (PMC5395462; doi:10.1002/ece3.2901)
Supplement: Supplementary file 1 [file ECE3-7-2846-s001.docx]

**Supporting Information**

**Models tested**

**TABLE S1** Models tested using the WDFW recreational time-series, REEF scuba surveys and WDFD trawl data. For each **Z** structure all combination of the u’s and all Q structures were tested. The two regions were North Puget Sound (NPS) and Puget Sound Proper (PSP); MCA = Marine Catch Area; GPS = Greater Puget Sound; All models included separate process variance for each rockfish trajectory with either no covariance between the trajectories or allowing covariance between all or some of the trajectories; see process covariance column with additional information in the footnotes and footnotes for Table S1. There were 71 observation processes: 54 Rec (9 MCA plus 6 regulatory time periods), 9 REEF, and 8 Trawl. Numbers in parenthesis are the number of estimated parameters for that term. For the trajectories, the estimated parameter is the initial state (*x*_0_). The number of *a* in **a** was 71 minus the number of population trajectories. A separate observation variance was estimated for each survey by MCA or trawl survey area. Thus 26 **R** parameters were estimated (Rec surveys in 9 MCAs, REEF surveys in 9 MCAs plus Trawl surveys in 8 areas).

| **Z** structures: Number and structure of pop. trajectories (**x**_0,_ **a**) | Growth rates (**u**) | Covariance structures (**Q**) |
| --- | --- | --- |
| Region x Survey (6, 65) | Region x Survey (6) | All trajectories covary (21) |
|  | Rec_NPS,_ Rec_PSP_, REEF, Trawl (4) | Rec_NPS_ & Rec_PSP_ covary (7) |
|  | Rec/Trawl + REEF (2) | Regions covary within surveys (9) |
|  | Survey (3) | No covariance (6) |
|  | Region (2) |  |
|  | GPS (1) |  |
| Rec_NPS_, Rec_PSP_,REEF, Trawl (4, 67) | Rec_NPS_, Rec_PSP_,REEF, Trawl (4) | All trajectories covary (10) |
|  | Rec/Trawl + REEF (2) | Rec_NPS_ & Rec_PSP_ covary (5) |
|  |  | No covariance (4) |
| RecNPS, RecPSP, REEF, Trawl (4, 67) | Survey (3) | All trajectories covary (10) |
|  | GPS (1) | Rec_NPS_ & Rec_PSP_ covary (5) |
|  |  | No covariance (4) |
| Rec_NPS_, Rec_PSP_, REEF, Trawl_NPS_, Trawl_PSP_ (5, 66) | Rec_NPS_, Rec_PSP_, REEF, Trawl_NPS_, Trawl_PSP_ (5) | All trajectories covary (15) |
|  | Rec, REEF, Trawl_NPS_, Trawl_PSP_ (4) | Rec and trawl btw regions (7) |
|  | Rec/Trawl + REEF (2) | Rec_NPS_ & Rec_PSP_ covary (6) |
|  | Survey (3) | No covariance (5) |
|  | GPS (1) |  |
| Rec/Trawl_NPS_, Rec/Trawl_PSP_, REEF_NPS_, REEF_PSP_ (4, 67) | Rec, REEF_NPS_, REEF_PSP_, Trawl (4) | All trajectories covary (10) |
|  | NPS, PSP, REEF_NPS_, REEF_PSP_ (4) | REEF_NPS_ & REEF_PSP_ covary (5) |
|  | Region (2) | No covariance (4) |
|  | GPS (1) |  |
| Rec/Trawl_NPS_, Rec/Trawl_PSP_, REEF (3, 68) | Rec/Trawl_NPS_, Rec/Trawl_PSP_, REEF (3) | All trajectories covary (6) |
|  | Rec/Trawl + REEF (2) | Rec/Trawl_NPS_ & Rec/Trawl_PSP_ covary (4) |
|  | GPS (1) | No covariance (3) |
| Survey (3, 68) | Survey (3) | All trajectories covary (6) |
|  | GPS (1) | No covariance (3) |
| Region (2, 69) | Region (2) | All trajectories covary (3) |
|  | GPS (1) | No covariance (2) |
| GPS (1, 70) | GPS (1) | No covariance (1) |

**Model selection and parameter estimates**

Using the recreational survey data alone to describe total rockfish assemblage dynamics, model selection identified two potential models with similar support (ΔAICc < 2.0, Table S1). Both models included separate rockfish trajectories for NPS and PSP and one overall Puget Sound-wide growth rate (*u*) but differed in whether or not they included process covariance between NPS and PSP. The model without covariance was unable to estimate process variance for PSP (Q_PSP_ = 0.000). Since we want to estimate process variance and since the model with covariance includes as a subset the model without covariance, we chose the model with covariance as the best model for the recreational data (Fig. 3a; Table 1 and S2).

Using the Rec and REEF survey data together, four models received similar support (Table S3). All four models included separate trajectories for the Rec data in NPS and PSP but one trajectory for the REEF data in all of Puget Sound. Two models, with no covariance in **Q**, were again unable to estimate process variance for the PSP Rec trajectory. We exclude these for the same reasons as above. Parameter estimates for the top two models are given in Tables S4 and S5.

The results using all three data sets were similar to the previous modeling exercises. The best models (Figs. 3d,e; Table S6) included four trajectories (Rec_NPS_, Rec_PSP_, REEF and Trawl), with again separate NPS and PSP trajectories for the Rec survey. The top model without covariance was again unable to estimate process variance for the PSP recreational trajectory (Table S6). Parameter estimates for the top two models are given in Tables S7 and S8.

Overall, there was concurrence across the analyses concerning the spatial structure of the population trajectories; eight of the top ten models had the following population trajectory structure: Rec_NPS_, Rec_PSP_, REEF, Trawl (Tables S1, S3 and S6), which means separate trajectories for Rec in NPS and PSP, and separate all-GPS REEF and Trawl observations. We did not find support for population structure at the MCA scale in the Rec-only analyses (Table S1) or in the combined Rec + REEF analyses (Table S3). In addition, there was concurrence regarding the covariance structure. Nine of the top ten models included covariance between Rec_NPS_ and Rec_PSP_ trajectories. Additionally, in the models that included NPS and PSP Trawl trajectories (Rec_NPS_, Rec_PSP_, REEF, Trawl_NPS_, Trawl_PSP_), the best models included covariance between the Trawl_NPS_ and Trawl_PSP_ trajectories. Only two of the top ten included covariance between the REEF trajectory and the Rec or Trawl trajectories. We see the same support for covariance within survey types in NPS and PSP when we look at only the models with a ‘Region x Survey’ trajectory structures. This structure allows six trajectories, one for each survey type and region (Table S9). In contrast, there was not as strong concurrence on the growth rate structure. Models with one shared growth rate (GPS) and models with separate growth rates for REEF, or separate growth rates for each survey type or each region, all had support. However the models with greatest support had a shared growth rate for the Rec and Trawl trajectories and a separate growth rate for the REEF trajectory.

**TABLE S2** Model selection criteria for MARSS models with using the WDFW recreational survey data only. AICc = Akiake’s Information Criterion corrected for sample size (Burnham & Anderson 1998). In columns 1 and 2, Region = 2 trajectories or growth rates for NPS and PSP, MCA = trajectories or growth rates for each of the 9 MCAs, and GPS = one Greater Puget Sound trajectory or growth rate. Cov = covariance between all population trajectories, No cov = no covariance among trajectories. Separate process variance was estimated for each trajectory. There were 54 observation time series. The number of estimated parameters for each term is in parentheses. For column 1, this is the number of initial states (**x**_0_) and number of *a* in **a**. There were 9 **R** parameters for each model.

| Number and structure of pop. trajectories (**x**_0,_ **a**) | Growth rates  (**u**) | Covariance structure (**Q**) | ΔAICc | Number of parameters  (**u**, **a**, **Q**, **R**, **x**_0_) |
| --- | --- | --- | --- | --- |
| Region (2, 52) | GPS (1) | Cov (3) | 0 | 67 |
| Region (2, 52)^1^ | GPS (1) | No cov. (2) | 0.2 | 66 |
| Region (2, 52) | Region (2) | Cov (3) | 2.98 | 68 |
| Region (2, 52) | Region (2) | No cov. (2) | 3.18 | 67 |
| GPS (1, 53) | GPS (1) | n/a (1) | 4.24 | 65 |
| MCA (9, 45) | GPS (1) | No cov. (9) | 31.66 | 73 |
| MCA (9, 45) | Region (2) | No cov. (9) | 33.82 | 74 |
| MCA (9, 45) | MCA (9) | No cov. (9) | 45.86 | 81 |
| MCA (9, 45) | GPS (1) | Cov (45) | 121.11 | 109 |
| MCA (9, 45) | Region (2) | Cov (45) | 125.4 | 110 |
| MCA (9, 45) | MCA (9) | Cov (45) | 147.29 | 117 |

^1^This model was unable to estimate process variance for PSP and was dropped from consideration.

**TABLE S3** Model results for the one *u* (growth rate)*,* two regions (NPS and PSP) model allowing covariance between NPS and PSP trajectories and using the recreational data only. Q = process variance, R = observation variance by Marine Conservation Area, NPS = North Puget Sound, PSP = Puget Sound Proper, GPS = Greater Puget Sound, CL = confidence limit.

| Parameter | Estimate | Lower 95% CL | Upper 95% CL |
| --- | --- | --- | --- |
| *u*_GPS_ | -0.038 | -0.051 | -0.021 |
| Q_NPS_ | 0.021 | 2.84E-08 | 0.025 |
| Q_PSP_ | 0.001 | 4.40E-08 | 0.009 |
| Q_NPS:PSP_ | 0.006 | 1.58E-10 | 0.013 |
| R_5_ | 0.064 | 0.024 | 0.078 |
| R_6_ | 0.168 | 0.071 | 0.184 |
| R_7_ | 0.015 | 0.005 | 0.022 |
| R_8_ | 0.087 | 0.039 | 0.106 |
| R_9_ | 0.159 | 0.067 | 0.167 |
| R_10_ | 0.200 | 0.086 | 0.23 |
| R_11_ | 0.149 | 0.074 | 0.189 |
| R_12_ | 0.202 | 0.072 | 0.233 |
| R_13_ | 0.239 | 0.109 | 0.253 |

**TABLE S4** Model selection criteria for MARSS models comparing different model structures using the WDFW recreational survey (Rec) and REEF survey. Model support was quantified using AICc (Akiake’s Information Criterion corrected for sample size). The geographic designations are North Puget Sound (NPS), Puget Sound Proper (PSP), MCA = management conservation area, and GPS = Greater Puget Sound. All models included separate process variance for each rockfish trajectory with either no covariance between the trajectories or allowing covariance between all or some of the trajectories; see process covariance column with additional information in the footnotes. There were 63 observation time series: 54 Rec (9 MCAs and 6 regulatory time periods) and 9 REEF (1 for each MCA). Numbers in parenthesis are the number of estimated parameters for term. For the population trajectories, the estimated parameters are the initial value of the trajectory (*x*_0_) and the scaling parameters (*a*). The number of *a* in **a** was 54 (the number of observation time series) minus the number of trajectories. A separate observation variance was estimated for each survey in each MCA. Thus 18 **R** parameters were estimated (one for each Rec survey in 9 MCAs plus one for each REEF survey in 9 MCAs).

| Number and structure of trajectories (**x**_0,_ **a**)^2^ | Growth rates (**u**)^3^ | | Rockfish trajectory covariance structure (**Q**)^4^ | | ΔAICc | | Number of parameters  (**u**, **a**, **Q**, **R**, **x**_0_) |
| --- | --- | --- | --- | --- | --- | --- | --- |
| Rec_NPS_, Rec_PSP_, REEF (3, 60) | | Survey (2) | | Rec_NPS_ & Rec_PSP_ covary (4) | | 0.00 | 87 |
| Rec_NPS_, Rec_PSP_, REEF (3, 60)^1^ | | Survey (2) | | No covariance (3) | | 0.21 | 86 |
| Rec_NPS_, Rec_PSP_, REEF (3, 60)^1^ | | GPS (1) | | No covariance (3) | | 1.12 | 85 |
| Rec_NPS_, Rec_PSP_, REEF (3, 60) | | GPS (1) | | Rec_NPS_ & Rec_PSP_ covary (4) | | 1.40 | 86 |
| Rec_NPS_, Rec_PSP_, REEF (3, 60) | | Rec_NPS_, Rec_PSP_, REEF (3) | | Rec_NPS_ & Rec_PSP_ covary (4) | | 2.89 | 88 |
| Rec_NPS_, Rec_PSP_, REEF (3, 60) | | Rec_NPS_, Rec_PSP_, REEF (3) | | No covariance (3) | | 3.14 | 87 |
| Survey (2, 61) | | Survey (2) | | No covariance (2) | | 4.30 | 85 |
| Region x Survey (4, 59) | | Survey (2) | | Surveys covary within regions (6) | | 4.51 | 89 |
| Survey (2, 61) | | GPS (1) | | No covariance (2) | | 5.12 | 84 |
| Rec_NPS_, Rec_PSP_, REEF (3, 60) | | Survey (2) | | All trajectories covary (6) | | 5.43 | 89 |
| Region x Survey (4, 59) | | GPS (1) | | Surveys covary within regions (6) | | 5.73 | 88 |
| Rec_NPS_, Rec_PSP_, REEF (3, 60) | | GPS (1) | | All trajectories covary (6) | | 6.55 | 88 |
| Region x Survey (4, 59) | | Survey (2) | | Regions covary within surveys (6) | | 6.61 | 89 |
| Survey (2, 61) | | Survey (2) | | All trajectories covary (3) | | 7.17 | 86 |
| Survey (2, 61) | | GPS (1) | | All trajectories covary (3) | | 7.51 | 85 |
| Region x Survey (4, 59) | | Region x Survey (4) | | No covariance (4) | | 7.86 | 89 |
| Rec_NPS_, Rec_PSP_, REEF (3, 60) | | Rec_NPS_, Rec_PSP_, REEF (3) | | All trajectories covary (6) | | 8.06 | 90 |
| Region x Survey (4, 59) | | Region (2) | | Regions covary within surveys (6) | | 8.61 | 89 |
| Rec, REEF_NPS_, REEF_PSP_ (3, 60) | | Survey (2) | | REEF_NPS_ & REEF_PSP_ covary (4) | | 8.75 | 87 |
| Rec, REEF_NPS_, REEF_PSP_ (3, 60) | | Rec, REEF_NPS_, REEF_PSP_ (3) | | No covariance (3) | | 8.85 | 87 |
| Rec, REEF_NPS_, REEF_PSP_ (3, 60) | | GPS (1) | | REEF_NPS_ & REEF_PSP_ covary (4) | | 9.43 | 86 |
| Region x Survey (4, 59) | | GPS (1) | | No covariance (4) | | 9.70 | 86 |
| Region x Survey (4, 59) | | GPS (1) | | Regions covary within surveys (6) | | 9.70 | 88 |
| Region x Survey (4, 59) | | Region (2) | | No covariance (4) | | 10.03 | 87 |
| Region x Survey (4, 59) | | Region x Survey (4) | | Surveys covary within regions (6) | | 10.07 | 91 |
| Rec, REEF_NPS_, REEF_PSP_ (3, 60) | | Survey (2) | | All trajectories covary (6) | | 11.02 | 89 |
| Rec, REEF_NPS_, REEF_PSP_ (3, 60) | | GPS (1) | | No covariance (3) | | 11.05 | 85 |
| Rec, REEF_NPS_, REEF_PSP_ (3, 60) | | Rec, REEF_NPS_, REEF_PSP_ (3) | | REEF_NPS_ & REEF_PSP_ covary (4) | | 11.34 | 88 |
| Region x Survey (4, 59) | | Region (2) | | Surveys covary within regions (6) | | 11.95 | 89 |
| Region x Survey (4, 59) | | Region x Survey (4) | | Regions covary within surveys (6) | | 12.20 | 91 |
| Rec, REEF_NPS_, REEF_PSP_ (3, 60) | | Rec, REEF_NPS_, REEF_PSP_ (3) | | All trajectories covary (6) | | 13.14 | 90 |
| Rec, REEF_NPS_, REEF_PSP_ (3, 60) | | GPS (1) | | All trajectories covary (6) | | 15.43 | 88 |
| Region x Survey (4, 59) | | Survey (2) | | All trajectories covary (10) | | 15.97 | 93 |
| Region x Survey (4, 59) | | GPS (1) | | All trajectories covary (10) | | 16.98 | 92 |
| Region x Survey (4, 59) | | Region (2) | | All trajectories covary (10) | | 20.57 | 93 |
| Region x Survey (4, 59) | | Region x Survey (4) | | All trajectories covary (10) | | 21.14 | 95 |
| Region (2, 61) | | GPS (1) | | No covariance (2) | | 22.99 | 84 |
| Region (2, 61) | | GPS (1) | | All trajectories covary (10) | | 23.93 | 85 |
| GPS (1, 62) | | GPS (1) | | No covariance (2) | | 24.22 | 83 |
| Region (2, 61) | | Region (2) | | No covariance (2) | | 25.41 | 85 |
| Region (2, 61) | | Region (2) | | All trajectories covary (10) | | 26.24 | 86 |
| MCA x Survey (18, 45) | | Survey (2) | | No covariance (18) | | 66.21 | 102 |
| MCA x Survey (18, 45) | | Region x Survey (4) | | No covariance (18) | | 70.61 | 103 |
| MCA x Survey (18, 45) | | GPS (1) | | No covariance (18) | | 79.06 | 100 |
| MCA x Survey (18, 45) | | Region (2) | | No covariance (18) | | 86.34 | 101 |
| MCA x Survey (18, 45) | | MCA x Survey (18) | | No covariance (18) | | 96.41 | 117 |
| MCA x Survey (18, 45) | | Survey (2) | | All trajectories covary (171) | | 722.73 | 255 |
| MCA x Survey (18, 45) | | GPS (1) | | All trajectories covary (171) | | 725.58 | 253 |
| MCA x Survey (18, 45) | | Region x Survey (4) | | All trajectories covary (171) | | 733.34 | 256 |
| MCA x Survey (18, 45) | | Region (2) | | All trajectories covary (171) | | 733.57 | 254 |
| MCA x Survey (18, 45) | | MCA x Survey (18) | | All trajectories covary (171) | | 849.45 | 270 |

^1^These models were unable to estimate processes variance for Rec_PSP_ and were dropped from consideration.

^2^The rockfish trajectory column shows the number and structure of the trajectories. The structure is as labeled. For example, Rec_NPS_, Rec_PSP_, REEF = 3 trajectories for each of these observation types; all Rec observations in NPS observe the Rec_NPS_ trajectory, all Rec observations in PSP observe the Rec_PSP_ trajectory, and all REEF observations are observing the REEF trajectory. In addition, the following abbreviations are used. Survey = one trajectory for each survey type (Rec or REEF). Region x Survey = Rec_NPS_, Rec_PSP_, REEF_NPS_, and REEF_PSP_ trajectories. MCA x Survey = Each survey in each MCA observes an different trajectory. Thus there are 9 x 2 trajectories. GPS = a single trajectory and all observations are of this one trajectory.

^3^The growth rate column shows the number of the growth rates. These are analogous to the rockfish trajectory column. Thus, Region x Survey = separate growth rates estimated for Rec_NPS_, Rec_PSP_, REEF_NPS_, and REEF_PSP_. GPS = a single growth rate for all trajectories.

^4^ The covariance column shows which covariances were estimated between the trajectories. No covariance: trajectories were treated as independent and a diagonal variance-covariance matrix was assumed. One variance estimated for each trajectory. Rec_NPS_ & Rec_PSP_ covary = covariance estimated between the Rec_NPS_ and Rec_PSP_  trajectories but set to 0 between Rec and all other trajectories. Regions covary within surveys = covariance estimated between surveys in each region thus there are Rec_NPS_:Rec_PSP_ and a REEF_NPS_:REEF_PSP_ covariance terms. No covariance terms between surveys (e.g., Rec_NPS_:REEF covariance term set to 0). All trajectories covary = an unconstrained variance-covariance matrix is estimated. The size of the matrix is determined by the number of trajectories. Thus if 18 trajectories are estimated, the matrix is 18x18 and there are 18 variances and 153 covariances. Surveys covary within regions = covariance is estimated between the surveys within the same region. Thus Rec_NPS_:REEF_NPS_ and Rec_PSP_:REEF_PSP_ covariance terms are estimated. No covariance terms between surveys in different regions, thus the Rec_NPS_:Rec_PSP_ covariance term is set to 0.

**TABLE S5** Parameter estimates for the one *u* rate, three rockfish trajectories (Rec_NPS_, Rec_PSP_ and REEF) model allowing covariance between the Rec_NPS_ and Rec_PSP_ trajectories. This model used the WDFW recreational survey (Rec) and the REEF survey. *u* = growth rate. A growth rate of -0.031 translates to approximately a 3.1% per year decline. Q = process variance and covariance. There was one process variance estimated for each of Rec_NPS_, Rec_PSP_ and REEF plus a covariance term between the Rec trajectories (Q_Rec NPS:Rec PSP_). R = observation variance by survey type and Marine Conservation Area. R_Rec 10_ is the observation variance for the Rec survey in MCA 10. The geographic areas are NPS = North Puget Sound, PSP = Puget Sound Proper, GPS = Greater Puget Sound. CL = confidence limit. CLs were calculated using the estimated Hessian calculation provided in the MARSS R package.

| Parameter | Estimate | Lower 95% CL | Upper 95% CL | |
| --- | --- | --- | --- | --- |
| *u*_GPS_ | -0.031 | -0.049 | -0.013 | |
| Q_Rec NPS_ | 0.021 | 7.5 x10^-8^ | 0.028 | |
| Q_Rec PSP_ | 0.001 | 1.0 x10^-7^ | 0.009 | |
| Q_Rec NPS:Rec PSP_ | 0.005 | -0.001 | 0.014 | |
| Q_REEF_ | 0.026 | 9.5 x10^-8^ | 0.042 | |
| R_Rec 5_ | 0.063 | 0.026 | 0.085 | |
| R_Rec 6_ | 0.168 | 0.075 | 0.170 | |
| R_Rec 7_ | 0.015 | 0.005 | 0.024 | |
| R_Rec 8_ | 0.532 | 0.112 | 0.876 | |
| R_Rec 9_ | 0.233 | 0.108 | 0.323 | |
| R_Rec 10_ | 0.166 | 0.059 | 0.252 |  |
| R_Rec 11_ | 0.006 | 6.0 x10^-6^ | 0.019 |  |
| R_Rec 12_ | 0.070 | 0.026 | 0.121 |  |
| R_Rec 13_ | 0.244 | 0.097 | 0.389 |  |
| R_REEF 5_ | 0.713 | 0.175 | 1.381 |  |
| R_REEF 6_ | 1.789 | 0.542 | 2.646 |  |
| R_REEF 7_ | 0.160 | 0.056 | 0.225 |  |
| R_REEF 8_ | 0.532 | 0.112 | 0.876 |  |
| R_REEF 9_ | 0.233 | 0.108 | 0.323 |  |
| R_REEF 10_ | 0.166 | 0.059 | 0.252 |  |
| R_REEF 11_ | 0.006 | 6.0 x10^-6^ | 0.019 |  |
| R_REEF 12_ | 0.070 | 0.026 | 0.121 |  |
| R_REEF 13_ | 0.244 | 0.097 | 0.389 |  |

**TABLE S6** Model results for the model with two *u* (different growth rates for the Rec versus REEF trajectories) and three states (Rec_NPS_, Rec_PSP_ and REEF). See Table S2 for explanations of the labels.

| Parameter | Estimate | Lower 95% CL | Upper 95% CL |
| --- | --- | --- | --- |
| *u*_Rec_ | -0.039 | -0.056 | -0.022 |
| *u*_REEF_ | 0.041 | -0.005 | 0.109 |
| Q_Rec NPS_ | 0.017 | 5.74E-08 | 0.030 |
| Q_Rec PSP_ | 0.002 | 8.98E-08 | 0.008 |
| Q_Rec NPS:Rec PSP_ | 0.006 | -1.79E-04 | 0.015 |
| Q_REEF_ | 0.012 | 5.02E-08 | 0.031 |
| R_Rec 5_ | 0.057 | 0.028 | 0.072 |
| R_Rec 6_ | 0.145 | 0.080 | 0.187 |
| R_Rec 7_ | 0.014 | 0.003 | 0.024 |
| R_Rec 8_ | 0.079 | 0.038 | 0.103 |
| R_Rec 9_ | 0.137 | 0.074 | 0.170 |
| R_Rec 10_ | 0.171 | 0.094 | 0.218 |
| R_Rec 11_ | 0.135 | 0.070 | 0.179 |
| R_Rec 12_ | 0.170 | 0.069 | 0.203 |
| R_Rec 13_ | 0.206 | 0.114 | 0.256 |
| R_REEF 5_ | 0.679 | 0.246 | 1.335 |
| R_REEF 6_ | 1.547 | 0.471 | 3.120 |
| R_REEF 7_ | 0.147 | 0.064 | 0.246 |
| R_REEF 8_ | 0.481 | 0.128 | 0.814 |
| R_REEF 9_ | 0.225 | 0.097 | 0.408 |
| R_REEF 10_ | 0.145 | 0.064 | 0.260 |
| R_REEF 11_ | 0.008 | 3.34E-05 | 0.024 |
| R_REEF 12_ | 0.066 | 0.027 | 0.118 |
| R_REEF 13_ | 0.235 | 0.086 | 0.381 |

**TABLE S7** Model selection criteria for MARSS models comparing different model structures using the WDFW recreational survey (Rec), REEF survey and WDFW trawl survey (Trawl). Data support for models was quantified using AICc (Akiake’s Information Criterion corrected for sample size). The two regions were North Puget Sound (NPS) and Puget Sound Proper (PSP); MCA = Marine Catch Area; GPS = Greater Puget Sound; All models included separate process variance for each rockfish trajectory with either no covariance between the trajectories or allowing covariance between all or some of the trajectories; see process covariance column with additional information in the footnotes and footnotes for Table S1. There were 71 observation processes: 54 Rec (9 MCA plus 6 regulatory time periods), 9 REEF, and 8 Trawl. Numbers in parenthesis are the number of estimated parameters for that term. For the trajectories, the estimated parameter is the initial state (*x*_0_). The number of *a* in **a** was 71 minus the number of population trajectories. A separate observation variance was estimated for each survey by MCA or trawl survey area. Thus 26 **R** parameters were estimated (Rec surveys in 9 MCAs, REEF surveys in 9 MCAs plus Trawl surveys in 8 areas).

| Number and structure of trajectories  (**x**_0_, **a**) ^2^ | | Growth rates (**u**) ^3^ | | Rockfish trajectory covariance structure (**Q**) ^4^ | | ΔAICc | | Number of parameters  (**u**, **a**, **Q**, **R**, **x**_0_) | |  |
| --- | --- | --- | --- | --- | --- | --- | --- | --- | --- | --- |
| Rec_NPS_, Rec_PSP_,REEF, Trawl (4, 67) | Rec/Trawl + REEF (2) | | Rec_NPS_ & Rec_PSP_ covary (5) | | 0.00 | | 104 | |  |  |
| Rec_NPS_, Rec_PSP_,REEF, Trawl (4, 67)^1^ | GPS (1) | | No covariance (4) | | 1.11 | | 102 | |  |  |
| Rec_NPS_, Rec_PSP_,REEF, Trawl (4, 67) | GPS (1) | | Rec_NPS_ & Rec_PSP_ covary (5) | | 1.39 | | 103 | |  |  |
| Rec_NPS_, Rec_PSP_, REEF, Trawl_NPS_, Trawl_PSP_ (5, 66) | Rec/Trawl + REEF (2) | | Rec and trawl btw regions (7) | | 1.96 | | 106 | |  | |
| Rec_NPS_, Rec_PSP_,REEF, Trawl (4, 67) | GPS (1) | | All trajectories covary (10) | | 2.71 | | 108 | |  |  |
| Rec_NPS_, Rec_PSP_,REEF, Trawl (4, 67) | Survey (3) | | Rec_NPS_ & Rec_PSP_ covary (5) | | 2.96 | | 105 | |  |  |
| Rec_NPS_, Rec_PSP_,REEF, Trawl (4, 67) | Rec/Trawl + REEF (2) | | All trajectories covary (10) | | 3.37 | | 109 | |  |  |
| Rec_NPS_, Rec_PSP_, REEF, Trawl_NPS_, Trawl_PSP_ (5, 66) | GPS (1) | | Rec and trawl btw regions (7) | | 3.67 | | 105 | |  |  |
| Rec_NPS_, Rec_PSP_,REEF, Trawl (4, 67) | Rec/Trawl + REEF (2) | | No covariance (4) | | 3.96 | | 103 | |  |  |
| Rec_NPS_, Rec_PSP_, REEF, Trawl_NPS_, Trawl_PSP_ (5, 66) | Survey (3) | | Rec and trawl btw regions (7) | | 4.66 | | 107 | |  |  |
| Survey (3, 68) | GPS (1) | | No covariance (3) | | 5.11 | | 101 | |  |  |
| Rec_NPS_, Rec_PSP_,REEF, Trawl (4, 67) | Rec_NPS_, Rec_PSP_,REEF, Trawl (4) | | Rec_NPS_ & Rec_PSP_ covary (5) | | 5.88 | | 106 | |  |  |
| Rec_NPS_, Rec_PSP_,REEF, Trawl (4, 67) | Rec_NPS_, Rec_PSP_,REEF, Trawl (4) | | No covariance (4) | | 6.10 | | 105 | |  |  |
| Region x Survey (6, 65) | Rec/Trawl + REEF (2) | | Regions covary within surveys (9) | | 6.54 | | 108 | |  |  |
| Rec_NPS,_ Rec_PSP_,REEF, Trawl (4, 67) | Survey (3) | | No covariance (4) | | 6.90 | | 104 | |  |  |
| Survey (3, 68) | Survey (3) | | No covariance (3) | | 7.21 | | 103 | |  |  |
| Rec_NPS_, Rec_PSP_, REEF, Trawl_NPS_, Trawl_PSP_ (5, 66) | Rec, REEF, Trawl_NPS_, Trawl_PSP_ (4) | | Rec and trawl btw regions (7) | | 7.51 | | 108 | |  |  |
| Region x Survey (6, 65) | GPS (1) | | Regions covary within surveys (9) | | 8.06 | | 107 | |  |  |
| Survey (3, 68) | GPS (1) | | All trajectories covary (6) | | 8.50 | | 104 | |  |  |
| Rec_NPS_, Rec_PSP_, REEF, Trawl_NPS_, Trawl_PSP_ (5, 66) | Rec/Trawl + REEF (2) | | Rec_NPS_ & Rec_PSP_ covary (6) | | 9.10 | | 105 | |  |  |
| Region x Survey (6, 65) | Survey (3) | | Regions covary within surveys (9) | | 9.26 | | 109 | |  |  |
| Rec_NPS_, Rec_PSP_,REEF, Trawl (4, 67) | Rec_NPS_, Rec_PSP_,REEF, Trawl (4) | | All trajectories covary (10) | | 9.48 | | 111 | |  |  |
| Rec_NPS_, Rec_PSP_, REEF, Trawl_NPS_, Trawl_PSP_ (5, 66) | Rec/Trawl + REEF (2) | | No covariance (5) | | 9.94 | | 104 | |  |  |
| Rec_NPS_, Rec_PSP_, REEF, Trawl_NPS_, Trawl_PSP_ (5, 66) | Rec_NPS_, Rec_PSP_, REEF, Trawl_NPS_, Trawl_PSP_ (5) | | Rec and trawl btw regions (7) | | 10.46 | | 109 | |  |  |
| Region x Survey (6, 65) | Region (2) | | Regions covary within surveys (9) | | 10.50 | | 108 | |  |  |
| Rec_NPS_, Rec_PSP_, REEF, Trawl_NPS_, Trawl_PSP_ (5, 66) | GPS (1) | | Rec_NPS_ & Rec_PSP_ covary (6) | | 11.07 | | 104 | |  |  |
| Rec_NPS_, Rec_PSP_, REEF, Trawl_NPS_, Trawl_PSP_ (5, 66) | GPS (1) | | No covariance (5) | | 11.38 | | 103 | |  |  |
| Rec_NPS_, Rec_PSP_, REEF, Trawl_NPS_, Trawl_PSP_ (5, 66) | Survey (3) | | Rec_NPS_ & Rec_PSP_ covary (6) | | 11.87 | | 106 | |  |  |
| Region x Survey (6, 65) | Rec/Trawl + REEF (2) | | No covariance (6) | | 11.98 | | 105 | |  |  |
| Rec_NPS_, Rec_PSP_, REEF, Trawl_NPS_, Trawl_PSP_ (5, 66) | Survey (3) | | No covariance (5) | | 12.14 | | 105 | |  |  |
| Survey (3, 68) | Survey (3) | | All trajectories covary (6) | | 12.36 | | 106 | |  |  |
| RecNPS, RecPSP, REEF, Trawl (4, 67) | Survey (3) | | All trajectories covary (10) | | 12.92 | | 110 | |  |  |
| Region x Survey (6, 65) | Survey (3) | | No covariance (6) | | 13.93 | | 106 | |  |  |
| Rec_NPS_, Rec_PSP_, REEF, Trawl_NPS_, Trawl_PSP_ (5, 66) | GPS (1) | | All trajectories covary (15) | | 14.22 | | 113 | |  |  |
| Rec/Trawl_NPS_, Rec/Trawl_PSP_, REEF (3, 68) | Rec/Trawl + REEF (2) | | Rec/Trawl_NPS_ & Rec/Trawl_PSP_ covary (4) | | 14.62 | | 103 | |  |  |
| Rec_NPS_, Rec_PSP_, REEF, Trawl_NPS_, Trawl_PSP_ (5, 66) | Rec, REEF, Trawl_NPS_, Trawl_PSP_ (4) | | Rec_NPS_ & Rec_PSP_ covary (6) | | 14.70 | | 107 | |  |  |
| Rec_NPS_, Rec_PSP_, REEF, Trawl_NPS_, Trawl_PSP_ (5, 66) | Rec/Trawl + REEF (2) | | All trajectories covary (15) | | 14.90 | | 114 | |  |  |
| Rec_NPS_, Rec_PSP_, REEF, Trawl_NPS_, Trawl_PSP_ (5, 66) | Rec, REEF, Trawl_NPS_, Trawl_PSP_ (4) | | No covariance (5) | | 14.96 | | 106 | |  |  |
| Rec/Trawl_NPS_, Rec/Trawl_PSP_, REEF (3, 68) | Rec/Trawl + REEF (2) | | No covariance (3) | | 15.45 | | 102 | |  |  |
| Rec/Trawl_NPS_, Rec/Trawl_PSP_, REEF (3, 68) | GPS (1) | | Rec/Trawl_NPS_ & Rec/Trawl_PSP_ covary (4) | | 16.60 | | 102 | |  |  |
| Rec/Trawl_NPS_, Rec/Trawl_PSP_, REEF (3, 68) | GPS (1) | | No covariance (3) | | 16.88 | | 101 | |  |  |
| Region x Survey (6, 65) | Rec_NPS_, Rec_PSP_, REEF, Trawl (4) | | No covariance (6) | | 16.96 | | 107 | |  |  |
| Rec/Trawl_NPS_, Rec/Trawl_PSP_, REEF (3, 68) | Rec/Trawl_NPS_, Rec/Trawl_PSP_, REEF (3) | | Rec/Trawl_NPS_ & Rec/Trawl_PSP_ covary (4) | | 17.22 | | 104 | |  |  |
| Rec_NPS_, Rec_PSP_, REEF, Trawl_NPS_, Trawl_PSP_ (5, 66) | Rec_NPS_, Rec_PSP_, REEF, Trawl_NPS_, Trawl_PSP_ (5) | | Rec_NPS_ & Rec_PSP_ covary (6) | | 17.64 | | 108 | |  |  |
| Region x Survey (6, 65) | Region x Survey (6) | | Regions covary within surveys (9) | | 17.80 | | 112 | |  |  |
| Rec/Trawl_NPS_, Rec/Trawl_PSP_, REEF (3, 68) | Rec/Trawl_NPS_, Rec/Trawl_PSP_, REEF (3) | | No covariance (3) | | 18.34 | | 103 | |  |  |
| Rec_NPS_, Rec_PSP_, REEF, Trawl_NPS_, Trawl_PSP_ (5, 66) | Survey (3) | | All trajectories covary (15) | | 18.40 | | 115 | |  |  |
| Rec/Trawl_NPS_, Rec/Trawl_PSP_, REEF (3, 68) | GPS (1) | | All trajectories covary (6) | | 18.54 | | 104 | |  |  |
| Rec/Trawl_NPS_, Rec/Trawl_PSP_, REEF (3, 68) | Rec/Trawl + REEF (2) | | All trajectories covary (6) | | 19.12 | | 105 | |  |  |
| Region x Survey (6, 65) | Region (2) | | No covariance (6) | | 20.39 | | 105 | |  |  |
| Region x Survey (6, 65) | GPS (1) | | No covariance (6) | | 20.51 | | 104 | |  |  |
| Rec_NPS_, Rec_PSP_, REEF, Trawl_NPS_, Trawl_PSP_ (5, 66) | Rec, REEF, Trawl_NPS_, Trawl_PSP_ (4) | | All trajectories covary (15) | | 20.93 | | 116 | |  |  |
| Rec/Trawl_NPS_, Rec/Trawl_PSP_, REEF (3, 68) | Rec/Trawl_NPS_, Rec/Trawl_PSP_, REEF (3) | | All trajectories covary (6) | | 21,82 | | 106 | |  |  |
| Region x Survey (6, 65) | Region x Survey (6) | | No covariance (6) | | 22.65 | | 109 | |  |  |
| Rec/Trawl_NPS_, Rec/Trawl_PSP_, REEF_NPS_, REEF_PSP_ (4, 67) | Rec, REEF_NPS_, REEF_PSP_, Trawl (4) | | No covariance (4) | | 23.02 | | 105 | |  |  |
| Rec_NPS_, Rec_PSP_, REEF, Trawl_NPS_, Trawl_PSP_ (5, 66) | Rec_NPS_, Rec_PSP_, REEF, Trawl_NPS_, Trawl_PSP_ (5) | | All trajectories covary (15) | | 24.01 | | 117 | |  |  |
| Rec/Trawl_NPS_, Rec/Trawl_PSP_, REEF_NPS_, REEF_PSP_ (4, 67) | Region (2) | | REEF_NPS_ & REEF_PSP_ covary (5) | | 25.27 | | 104 | |  |  |
| Rec/Trawl_NPS_, Rec/Trawl_PSP_, REEF_NPS_, REEF_PSP_ (4, 67) | Region (2) | | No covariance (4) | | 25.60 | | 103 | |  |  |
| Rec/Trawl_NPS_, Rec/Trawl_PSP_, REEF_NPS_, REEF_PSP_ (4, 67) | NPS, PSP, REEF_NPS_, REEF_PSP_ (4) | | REEF_NPS_ & REEF_PSP_ covary (5) | | 25.86 | | 106 | |  |  |
| Rec/Trawl_NPS_, Rec/Trawl_PSP_, REEF_NPS_, REEF_PSP_ (4, 67) | GPS (1) | | No covariance (4) | | 25.96 | | 102 | |  |  |
| Region x Survey (6, 65) | Region x Survey (6) | | Rec_NPS_ & Rec_PSP_ covary (7) | | 27.61 | | 108 | |  |  |
| Rec/Trawl_NPS_, Rec/Trawl_PSP_, REEF_NPS_, REEF_PSP_ (4, 67) | GPS (1) | | All trajectories covary (10) | | 29.98 | | 108 | |  |  |
| Region x Survey (6, 65) | Rec/Trawl + REEF (2) | | All trajectories covary (21) | | 30.54 | | 120 | |  |  |
| Rec/Trawl_NPS_, Rec/Trawl_PSP_, REEF_NPS_, REEF_PSP_ (4, 67) | Region (2) | | All trajectories covary (10) | | 33.14 | | 109 | |  |  |
| Region x Survey (6, 65) | GPS (1) | | All trajectories covary (21) | | 34.52 | | 119 | |  |  |
| Region x Survey (6, 65) | Survey (3) | | All trajectories covary (21) | | 34.57 | | 121 | |  |  |
| Rec/Trawl_NPS_, Rec/Trawl_PSP_, REEF_NPS_, REEF_PSP_ (4, 67) | Rec, REEF_NPS_, REEF_PSP_, Trawl (4) | | All trajectories covary (10) | | 35.60 | | 111 | |  |  |
| Region x Survey (6, 65) | Rec_NPS,_ Rec_PSP_, REEF, Trawl (4) | | All trajectories covary (21) | | 37.86 | | 122 | |  |  |
| Region x Survey (6, 65) | Region (2) | | All trajectories covary (21) | | 38.49 | | 120 | |  |  |
| Region x Survey (6, 65) | Region x Survey (6) | | All trajectories covary (21) | | 43.33 | | 124 | |  |  |
| Region (2, 69) | GPS (1) | | No covariance (2) | | 66.50 | | 100 | |  |  |
| Region (2, 69) | Region (2) | | No covariance (2) | | 68.95 | | 101 | |  |  |
| Region (2, 69) | GPS (1) | | All trajectories covary (3) | | 69.37 | | 101 | |  |  |
| Region (2, 69) | Region (2) | | All trajectories covary (3) | | 71.86 | | 102 | |  |  |
| GPS (1, 70) | GPS (1) | | No covariance (1) | | 85.52 | | 99 | |  |  |

^1^These models were unable to estimate processes variance for Rec_PSP_ and were dropped from consideration.

^2^The rockfish trajectory column gives the number and designation of the trajectories. For example, Rec_NPS_, Rec_PSP_, REEF, Trawl = 4 trajectories as labeled. All Rec observations in NPS are of the Rec_NPS_, all Rec observations in PSP are of Rec_PSP_, all REEF observations are of the REEF trajectory and all Trawl observations are of the Trawl trajectory. In addition, there are the following abbreviations. Survey = one trajectory for each survey type (Rec, REEF & Trawl). Region x Survey = Rec_NPS_, Rec_PSP_, REEF_NPS_, REEF_PSP_, Trawl_NPS_, and Trawl_PSP_ trajectories. GPS = a single trajectory and all observations are of this one trajectory.

^3^The growth rate column shows the number of the growth rates. These are analogous to the rockfish trajectory column (column 1). Thus, Region x Survey means that separate growth rates are estimated for Rec_NPS_, Rec_PSP_, REEF_NPS_, REEF_PSP_, Trawl_NPS_, and Trawl_PSP_. GPS = a single growth rate for all trajectories.

^4^The covariance column shows which covariances were estimated between the trajectories. No covariance = trajectories were treated as independent and a diagonal variance-covariance matrix was assumed. One variance estimated for each trajectory. Rec_NPS_ & Rec_PSP_ covary = covariance estimated between the Rec_NPS_ and Rec_PSP_  trajectories. All other covariances set to 0. Rec and trawl btw regions = covariance estimated between the Rec_NPS_ and Rec_PSP_  trajectories and Trawl_NPS_ and Trawl_PSP_  trajectories. All other covariances set to 0. Regions covary within surveys = Rec_NPS_:Rec_PSP_, REEF_NPS_:REEF_PSP_ and Trawl_NPS_:Trawl_PSP_ covariance terms are estimated. No covariance terms between surveys (e.g., Rec_NPS_:REEF covariance term set to 0). All trajectories covary = an unconstrained variance-covariance matrix is estimated. The size of the matrix is determined by the number of trajectories. Thus if 18 trajectories are estimated, the matrix is 18x18 and there are 18 variances and 153 covariances.

**TABLE S8** Model results for the two growth rates (combined Rec/Trawl *u*_Rec/Trawl_, separate REEF *u*_REEF)_, four rockfish trajectories (Rec_NPS_, Rec_PSP_, REEF, Trawl) model allowing covariance between the Rec_NPS_ and Rec_PSP_ trajectories. This model used all three data sets: the WDFW recreational survey (Rec), the REEF diver survey and the WDFW trawl survey (Trawl). The parameters are denoted as follows. Q_A_ = process variance of trajectory A, Q_A:B_ = process covariance between trajectories A and B, R_Rec#_ = observation variance for the Rec survey in Marine Conservation Area #, R_REEF#_ *=* observation variance for the REEF survey in Marine Conservation Area #, R_TrawlXX_*=* observation variance for the Trawl survey in area XX, *u* = growth rate where the number of *u* indicates the number of different u that were estimated. The geographic areas are NPS = North Puget Sound, PSP = Puget Sound Proper, GPS = Greater Puget Sound. See the legend of Figure S4 for the definitions of the geographic regions for the trawl surveys, e.g., R_Trawl GB_. CL = confidence limit. CLs were calculated using the estimated Hessian calculation provided in the MARSS R package.

| Parameter | Estimate | Lower 95% CL | Upper 95% CL |
| --- | --- | --- | --- |
| *u*_Rec/Trawl_ | -0.039 | -0.051 | -0.016 |
| *u*_REEF_ | 0.041 | -0.013 | 0.092 |
| Q_Rec NPS_ | 0.017 | 0.001 | 0.031 |
| Q_Rec PSP_ | 0.002 | -8 x 10-4 | 0.008 |
| Q_Rec NPS:Rec PSP_ | 0.006 | 2.5 x 10-4 | 0.012 |
| Q_REEF_ | 0.012 | 0.004 | 0.032 |
| Q_Trawl_ | 0.165 | 0.020 | 0.363 |
| R_Rec 5_ | 0.057 | 0.028 | 0.076 |
| R_Rec 6_ | 0.145 | 0.070 | 0.195 |
| R_Rec 7_ | 0.014 | 0.004 | 0.023 |
| R_Rec 8_ | 0.079 | 0.036 | 0.109 |
| R_Rec 9_ | 0.137 | 0.074 | 0.190 |
| R_Rec 10_ | 0.171 | 0.087 | 0.227 |
| R_Rec 11_ | 0.135 | 0.073 | 0.170 |
| R_Rec 12_ | 0.170 | 0.074 | 0.224 |
| R_Rec 13_ | 0.207 | 0.112 | 0.280 |
| R_REEF 5_ | 0.679 | 0.186 | 1.296 |
| R_REEF 6_ | 1.547 | 0.504 | 3.070 |
| R_REEF 7_ | 0.147 | 0.067 | 0.260 |
| R_REEF 8_ | 0.481 | 0.112 | 1.011 |
| R_REEF 9_ | 0.225 | 0.084 | 0.395 |
| R_REEF 10_ | 0.145 | 0.049 | 0.232 |
| R_REEF 11_ | 0.008 | -0.002 | 0.025 |
| R_REEF 12_ | 0.066 | 0.029 | 0.124 |
| R_REEF 13_ | 0.235 | 0.104 | 0.394 |
| R_Trawl GB_ | 0.242 | 0.060 | 0.444 |
| R_Trawl JE_ | 0.328 | 0.086 | 0.669 |
| R_Trawl JW_ | 0.241 | 0.045 | 0.550 |
| R_Trawl SJ_ | 0.240 | 0.039 | 0.520 |
| R_Trawl CS_ | 0.291 | 0.064 | 0.591 |
| R_Trawl HC_ | 0.204 | 0.056 | 0.396 |
| R_Trawl SS_ | 1.317 | 0.376 | 2.150 |
| R_Trawl WI_ | 0.250 | 0.035 | 0.477 |

**TABLE S9** Model results for the one growth rate (*u_GPS_*), four rockfish trajectories (Rec_NPS_, Rec_PSP_, REEF, Trawl) model allowing covariance between the Rec_NPS_ and Rec_PSP_ trajectories. This model used all three data sets: the WDFW recreational survey (Rec), the REEF diver survey and the WDFW trawl survey (Trawl). See Table S6 for key to parameter estimate abbreviations. CL = confidence limit. CLs were calculated using the estimated Hessian calculation provided in the MARSS R package.

| Parameter | Estimate | Lower 95% CL | Upper 95% CL |
| --- | --- | --- | --- |
| *u*_GPS_ | -0.031 | -0.050 | -0.013 |
| Q_Rec NPS_ | 0.020 | 7.3 x 10-8 | 0.027 |
| Q_Rec PSP_ | 0.001 | 1.1 x 10-7 | 0.007 |
| Q_Rec NPS:Rec PSP_ | 0.005 | -3.7 x 10-4 | 0.012 |
| Q_REEF_ | 0.024 | 0.001 | 0.041 |
| Q_Trawl_ | 0.172 | 0.013 | 0.343 |
| R_Rec 5_ | 0.062 | 0.030 | 0.076 |
| R_Rec 6_ | 0.157 | 0.082 | 0.195 |
| R_Rec 7_ | 0.015 | 0.005 | 0.021 |
| R_Rec 8_ | 0.090 | 0.045 | 0.103 |
| R_Rec 9_ | 0.158 | 0.070 | 0.177 |
| R_Rec 10_ | 0.191 | 0.086 | 0.229 |
| R_Rec 11_ | 0.153 | 0.068 | 0.170 |
| R_Rec 12_ | 0.215 | 0.075 | 0.202 |
| R_Rec 13_ | 0.242 | 0.106 | 0.246 |
| R_REEF 5_ | 0.708 | 0.237 | 1.160 |
| R_REEF 6_ | 1.686 | 0.451 | 2.793 |
| R_REEF 7_ | 0.156 | 0.057 | 0.246 |
| R_REEF 8_ | 0.535 | 0.102 | 0.892 |
| R_REEF 9_ | 0.236 | 0.099 | 0.370 |
| R_REEF 10_ | 0.157 | 0.059 | 0.268 |
| R_REEF 11_ | 0.006 | 5.8 x 10-6 | 0.025 |
| R_REEF 12_ | 0.070 | 0.024 | 0.118 |
| R_REEF 13_ | 0.254 | 0.072 | 0.455 |
| R_Trawl GB_ | 0.246 | 0.056 | 0.498 |
| R_Trawl JE_ | 0.352 | 0.065 | 0.658 |
| R_Trawl JW_ | 0.276 | 0.040 | 0.496 |
| R_Trawl SJ_ | 0.269 | 0.029 | 0.447 |
| R_Trawl CS_ | 0.333 | 0.065 | 0.470 |
| R_Trawl HC_ | 0.229 | 0.029 | 0.370 |
| R_Trawl SS_ | 1.457 | 0.413 | 2.639 |
| R_Trawl WI_ | 0.277 | 0.051 | 0.498 |

**TABLE S10** Model selection criteria for MARSS models using the WDFW recreational survey, REEF survey and WDFW trawl survey with only the ‘Region x Survey’ rockfish trajectory structure. This allows all regions-survey combination to have a separate trajectory. This model selection analysis allowed us to look at the support for *u* structure and trajectory covariance structure. See Table S3 for details on the columns.

| Number and structure of trajectories | Growth rates (*u*) | Rockfish trajectory covariance structure (Q) | ΔAICc |
| --- | --- | --- | --- |
| Region x Survey | Rec/Trawl + REEF (2) | Regions covary within surveys (9) | 0.00 |
| Region x Survey | GPS (1) | Regions covary within surveys (9) | 1.52 |
| Region x Survey | Survey (3) | Regions covary within surveys (9) | 2.72 |
| Region x Survey | Region (2) | Regions covary within surveys (9) | 3.96 |
| Region x Survey | Rec/Trawl + REEF (2) | No covariance (6) | 5.44 |
| Region x Survey | Survey (3) | No covariance (6) | 7.39 |
| Region x Survey | Rec/Trawl + REEF (2) | Surveys covary within regions (12) | 8.59 |
| Region x Survey | Rec_NPS_, Rec_PSP_, REEF, Trawl (4) | No covariance (6) | 10.42 |
| Region x Survey | Region x Survey (6) | Regions covary within surveys (9) | 11.36 |
| Region x Survey | Survey (3) | Surveys covary within regions (12) | 11.38 |
| Region x Survey | Region (2) | No covariance (6) | 13.85 |
| Region x Survey | GPS (1) | No covariance (6) | 13.97 |
| Region x Survey | Region x Survey (6) | No covariance (6) | 16.11 |
| Region x Survey | GPS (1) | Surveys covary within regions (12) | 19.03 |
| Region x Survey | Region x Survey (6) | Surveys covary within regions (12) | 20.19 |
| Region x Survey | Region x Survey (6) | Rec only covaries between regions (5) | 21.07 |
| Region x Survey | Region (2) | Surveys covary within regions (12) | 22.24 |
| Region x Survey | Rec/Trawl + REEF (2) | All trajectories covary (21) | 24.00 |
| Region x Survey | GPS (1) | All trajectories covary (21) | 27.98 |
| Region x Survey | Survey (3) | All trajectories covary (21) | 28.03 |
| Region x Survey | Rec_NPS_, Rec_PSP_, REEF, Trawl (4) | All trajectories covary (21) | 30.60 |
| Region x Survey | Region (2) | All trajectories covary (21) | 31.95 |
| Region x Survey | Region x Survey (6) | All trajectories covary (21) | 36.79 |

**FIGURE S1** Estimates of growth rate (*u*) from all models used in model selection (Tables S1, S3 and S6). The plot shows the range of estimates under different model parameterizations. The x-axis shows the type *u* estimate (2^nd^ column in the tables). Thus GPS on the x-axis shows the *u* estimates for models with a *u*_GPS_ term (one *u* for all of Puget Sound). Points have been jittered to prevent overlapping estimates. The red points show the *u* from the best-supported models (ΔAICc < 2).

**Supplement 2: Data descriptions**

*Data accessibility*

The data as used in the analyses can be found in:

Tonnes, D., Bhuthimethee, M., Sawchuck, J., Tolimieri, N., Andrews, K. & Nichols, K. (2016) Yelloweye rockfish (*Sebastes ruberrimus*), canary rockfish (*Sebastes pinniger*), and bocaccio (*Sebastes paucispinis*) of the Puget Sound/Georgia Basin: 5-Year Review: Summary and Evaluation. NOAA’s National Marine Fisheries Service, Office of Protected Resources, Seattle WA, USA.

WDFW Recreational Fishery Survey (1977-2014)

The WDFW Recreational Fishery Survey focuses on estimates of rockfish catch by boat-based anglers, who historically account for the majority of harvested rockfish (Palsson 2009). Boat-based anglers fish predominantly using rod and reel and can target rockfish at depths from several meters to over 120m in areas of high, rocky relief. Data up to 1994 relied heavily on seasonally limited angler information reported as part of the recreational salmon fisher surveys, and to a lesser degree on creel (dockside) surveys. After 1994, extensive closures of salmon fishing limited the catch information from many areas. In 2001, a new survey using phone and creel surveys was implemented to provide coverage for marine fisheries targeting diverse fish types (salmon, halibut, bottomfish) throughout the entire year and this replaced the previous survey in 2004.

The data used in the analysis can be found in Palsson (1988) and Palsson et al. (2009) for 1977 - 2007 and are summarized in Drake *et al*. (2010). WDFW provided additional data for 2008-2014 (Kraig 2015). While some data exist for 1965-1973 (Buckley 1967, Buckley 1968, Buckley 1970, Bargmann 1977), they are highly variable and were not broken out by MCA. These early data were not used in our analysis.

The raw data are shown in Fig. 4 (main article). The data are presented as the log of catch (both retained and released) per angler effort (CPUE), where effort is one angler trip. The data are for total rockfishes in nine Marine Catch Areas (MCA) in Puget Sound. North Puget Sound (NPS) = MCA 5-7. Puget Sound Proper (PSP) = MCA 8-13. The symbols in Figure 3 represent different bag limits (number of rockfish allowed to be retained by the angler) for the recreational fishery. Solid circles: early data that were not used because the data were not separated by MCA. Open squares: 1977-1982 no bag limits. Open circles: 1983-1993 bag limit of 10 in NPS and 5 in PSP. Open triangles: 1994-1999 bag limit of 5 in NPS and 3 in PSP. Open diamonds: 2000-2007 bag limit of 1 in all areas except where the bag limit was lowered to zero in MCA 12 in 2004 and beyond) and in 2001 retention of yelloweye and canary rockfish prohibited. Post 1994 also corresponds to the time period after widespread closure of the salmon fishery, which led to reduced catch information for many MCAs. Crosses (x): 2001-2009 same regulations as the open diamonds but using the new method for computing recreational CPUE that incorporated phone interview data. Pluses (+): 2010-2014 bag limit of 0 in all areas except MCA 5 where catch of black rockfish and blue rockfish is allowed and no bottom-fishing below 120 feet, and the new method for computing recreational CPUE. Note that the recreational CPUE includes ‘releases’ thus even when the bag limit is 0, the recreational CPUE is not zero (Fig. S4).

The steep declines in CPUE when the bag limit was decreased can be seen in 2000 when the bag limit went to 1 in all areas. It is also apparent that the new method of computing CPUE, which includes additional information from phone interviews, is scaled upward relative to the old method. The *a* scaling terms in the MARSS models allow us to model these step changes.

REEF Recreational Scuba Diver Surveys (1998-2014)

Puget Sound data were available for 1998-2014 (Fig. 5, main article) from the REEF recreational scuba diver survey (REEF 2014). The data are reported in abundance categories: single = single fish, few = 2–10 fish, many = 11–100 fish, and abundant = 101+ fish. Following Drake *et al*. (2010), we converted these abundance categories to minimum values (1, 2, 11 or 101 fishes). We then averaged observations by site within years to control for more frequent surveys at popular dive sites, and we only included dives from hard-bottom sites. We calculated a yearly mean number of total rockfish observed per dive for each MCA, but we excluded Puget Sound Rockfish, *S. emphaeus*, from the analyses. Puget Sound Rockfish are much smaller than other rockfishes, can occur in very high abundance ephemerally, and are not caught in the recreational fishery. We also excluded young-of-year fishes. These exclusions were done so that our REEF survey data would track the adult rockfish as did the recreational and trawl surveys.

WDFW Trawl Survey (1987-2014)

WDFW has conducted a fishery-independent trawl survey since 1987 throughout the Puget Sound (Fig. 6, main article). The survey is depth-stratified: 5-20, 21-40, 41-60, 60-120, and 121+ fathoms. See Palsson *et al*. (2009) for details on the survey methods prior to 2008. Effort is allocated among nine regions: central Puget Sound (CS), U.S. Strait of Georgia (GB), Hood Canal (HC), east U.S. Juan de Fuca (JE), west U.S. Juan de Fuca (JW), U.S. San Juan Archipelago (SJ), South Puget Sound (SS), and the Whidbey Basin (WI) (Figure 6). Prior to 2008 coverage ranged from 0-4 regions annually, with 4 regions being sampled only in 6 years, and the goal of the survey was to generate region-specific estimates of biomass. Beginning in 2008 the goal of the study shifted to providing Puget Sound-wide estimates of biomass for each species encountered, and every region has been sampled annually since. This shift in survey design greatly expanded effort geographically but required a shift to occupation of pre-selected index stations as opposed to randomly selected sites (Drake *et al*. 2010 Table 9), but has sampled pre-determined index stations in each region annually since 2008. R. Pacunski provided a complete download of the trawl data set in November of 2014.

Here we used data for the U.S. waters only: JE, JW, SJ, GS, HC, CS, SS, and WB. We did not include DB because there were only two data points across all years. For the current analysis, CPUE (number per m^2^) for total rockfishes was recalculated from the raw data file by dividing the number of individuals by the swept area of the trawl. We then calculated the mean CPUE for each year and for the eight regions listed above.

Species Composition Data

Species composition data exist for 1965-1973 (Buckley 1967, Buckley 1968, Buckley 1970, Bargmann 1977) and from the WDFW recreational survey (Palsson 1988, Palsson 2009, Kraig 2015). The identifications are done by WDFW staff as part of the creel survey of anglers’ retained catch, but for released catch anglers are relied upon to identify fish independently. Research has shown that estimates of species composition for these angler-identified fish are subject to significant bias and error (Sawchuk 2012).

For the years 1965 to 2007, we took species composition information directly from Drake *et al*. (2010) for bocaccio (Tables 11 and 12), canary (Tables 14 and 15) and yelloweye (Tables 17 and 18) rockfish; the Drake *et al*. 2010 data come from the citations above. For 1965 to 1979, Drake *et al*. 2010 do not give sampling effort. To calculate a decadal average, we calculated the mean of all values for each species in these years using the data in the aforementioned tables, but did not calculate 95% confidence limits because there was no information on sampling effort. For 1980 to 2007, the tables give the number of individual fish identified. We first converted percent to numbers of boccacio, canary and yelloweye rockfish and then summed the counts and total number identified by decade. We then estimated 95% binomial confidence limits using Wilson intervals (Agresti & Coull 1998) with the function binconf in the Hmisc package for R v3.2.4 (R Core Team 2016). For 2008-2014 we used the proportions reported by WDFW, and computed the numbers of individuals sampled from the total retained catch reported for the creel portion of the WDFW recreational survey. We did not use information from the phone survey portion of the survey or from discarded fish to ensure that trained personnel did the species identification. We then summed counts by decade and calculated the proportion of the assemblage represented by each species. As above, we used Wilson intervals to estimate binomial 95% confidence limits.

Species composition information is also reported in the REEF survey. However, canary and yelloweye rockfishes made up minor proportions (< 1%) of the rockfish observed by REEF divers (Fig. S2; Table S10) and were not reported in the initial years of the survey. The initial absence of both canary and yelloweye from 1998-2000 may have been due to diver learning and not an actual absence of these species. Both increased as a proportion of the rockfish assemblage from 2000 through approximately 2005-2010 and then decreased in prevalence. Divers observed bocaccio only twice on more than 11,000 dives. In the WDFW trawl survey, the three species were observed only rarely and it is not possible to infer any trends from the data (Fig. S3).

**Literature Cited**

Agresti, A. & Coull, B.A. (1998) Approximate is better than "exact" for interval estimation of binomial proportions. American Statistician **52**, 119-126.

Bargmann, G.G. (1977) The recreational hook and line fishery for marine fish in Puget Sound, 1968– 1973. WDFW Progress Rep. 33. Washington Dept. Fish and Wildlife, Olympia.

Buckley, R.M. (1967) 1965 bottomfish sport fishery. Washington Dept. Washington Dept. Fisheries Supplemental Progress Rep. Washington Dept. Fisheries, Olympia.

Buckley, R.M. (1968) 1966 bottomfish sport fishery occurring in Washington marine punch card areas 2 through 12. Washington Dept. Fisheries Supplemental Progress Rep. Washington Dept. Fisheries, Olympia., Washington Dept. Fisheries Supplemental Progress Rep. Washington Dept. Fisheries, Olympia.

Buckley, R.M. (1970) 1967 bottomfish sport fishery. Washington Dept. Fisheries Supplemental Progress Rep. Washington Dept. Fisheries, Olympia.

Burnham, K.P., & Anderson, D.R. (1998) *Model Selection and Inference: A Practical Information-theoretic Approach*. Springer-Verlag, New York, NY.

Drake, J., Berntson, E.A., Cope, J.M., Gustafson, R.G., Holmes, E.E., Levin, P.S., Tolimieri, N., Waples, R.S., Sogard, S., & Williams, G.D. (2010) Status review of five rockfish species in Puget Sound, Washington: Bocaccio (*Sebastes paucispinis*), canary rockfish (*S. pinniger*), yelloweye rockfish (*S. ruberrimus*), greenstriped rockfish (*S. elongatus*), and redstripe rockfish (*S. proriger*). U.S. Dept. of Commerce, NOAA Technical Memorandum, NMFS-NWFSC-108.

Kraig, E. (2015) Washington State Sport Catch Report 2012. pp. 83. Washington Department of Fish and Wildlife, Olympia, Washington.

Palsson, W.A. (1988) Bottomfish catch and effort statistics from boat-based recreational fisheries in Puget Sound, 1970–1985 (revised). Progress Rep. 261, Washington Dept. Fisheries, Olympia.

Palsson, W.A., Tsou, T.-S., Bargmann, G.G., Buckley, R.M., West, J.E., Mills, M.L., Cheng, Y.W., & Pacunski, R.E. (2009) The biology and assessment of rockfishes in Puget Sound. FPT 09-04. Washington Dept. Fish and Wildlife, Olympia.

R Core Team (2016) *R: A Language and Environment for Statistical Computing*. R Foundation for Statistical Computing, Vienna, Austria.


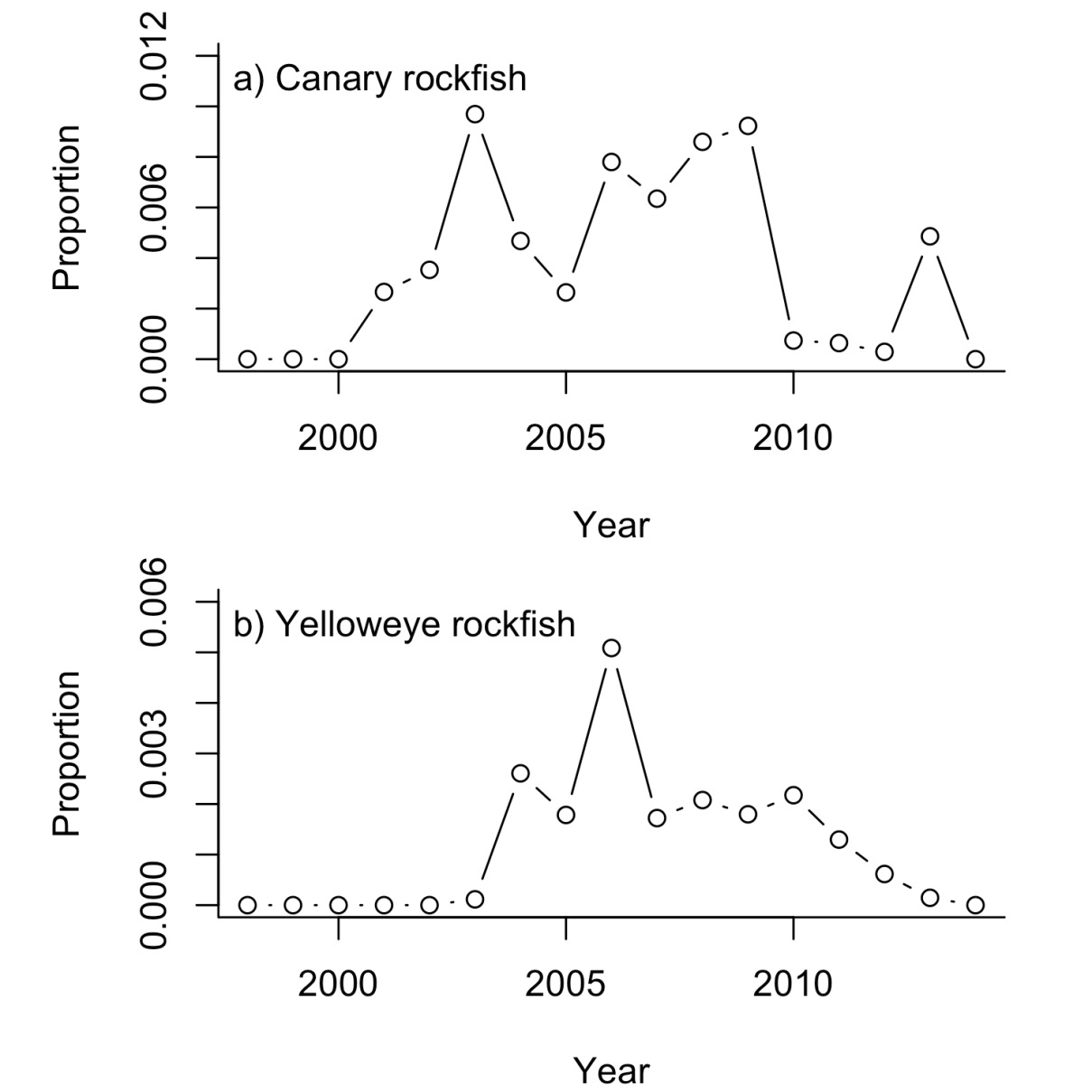


**FIGURE S2** Prevalence of a) canary and b) yelloweye rockfish as a proportion of the total rockfish assemblage from the REEF surveys for Greater Puget Sound (all MCAs). Bocaccio were recorded only twice out of more than 11,000 dives and are not shown.


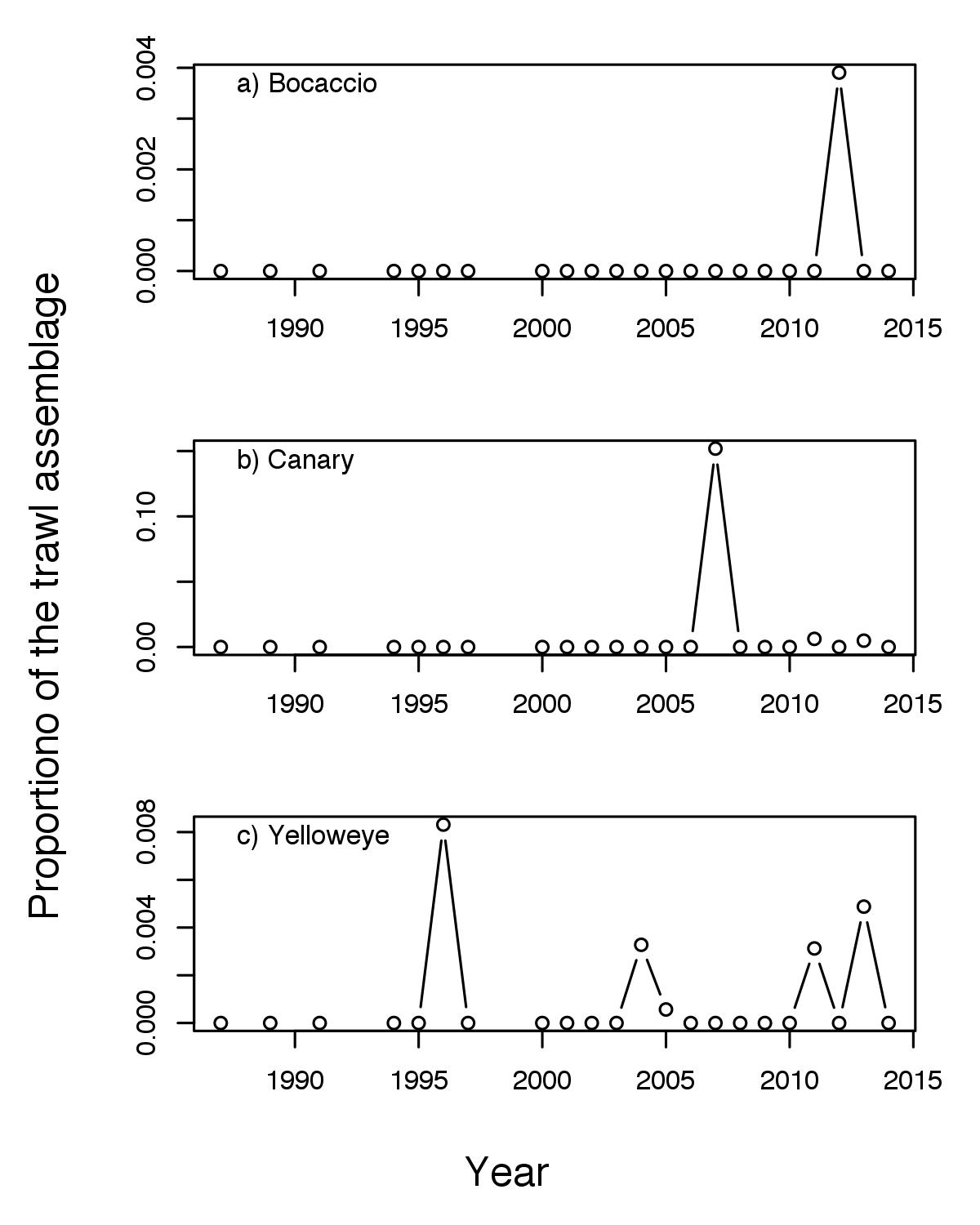


**Figure S3** Prevalence of a) bocaccio, b) canary and c) yelloweye rockfish as a proportion of the total rockfish assemblage in the WDFW trawl survey. Yelloweye 1990-1999 average = 0.0016, 2000-2009 average = 0.0005, 2010-2014 average = 0.0014.


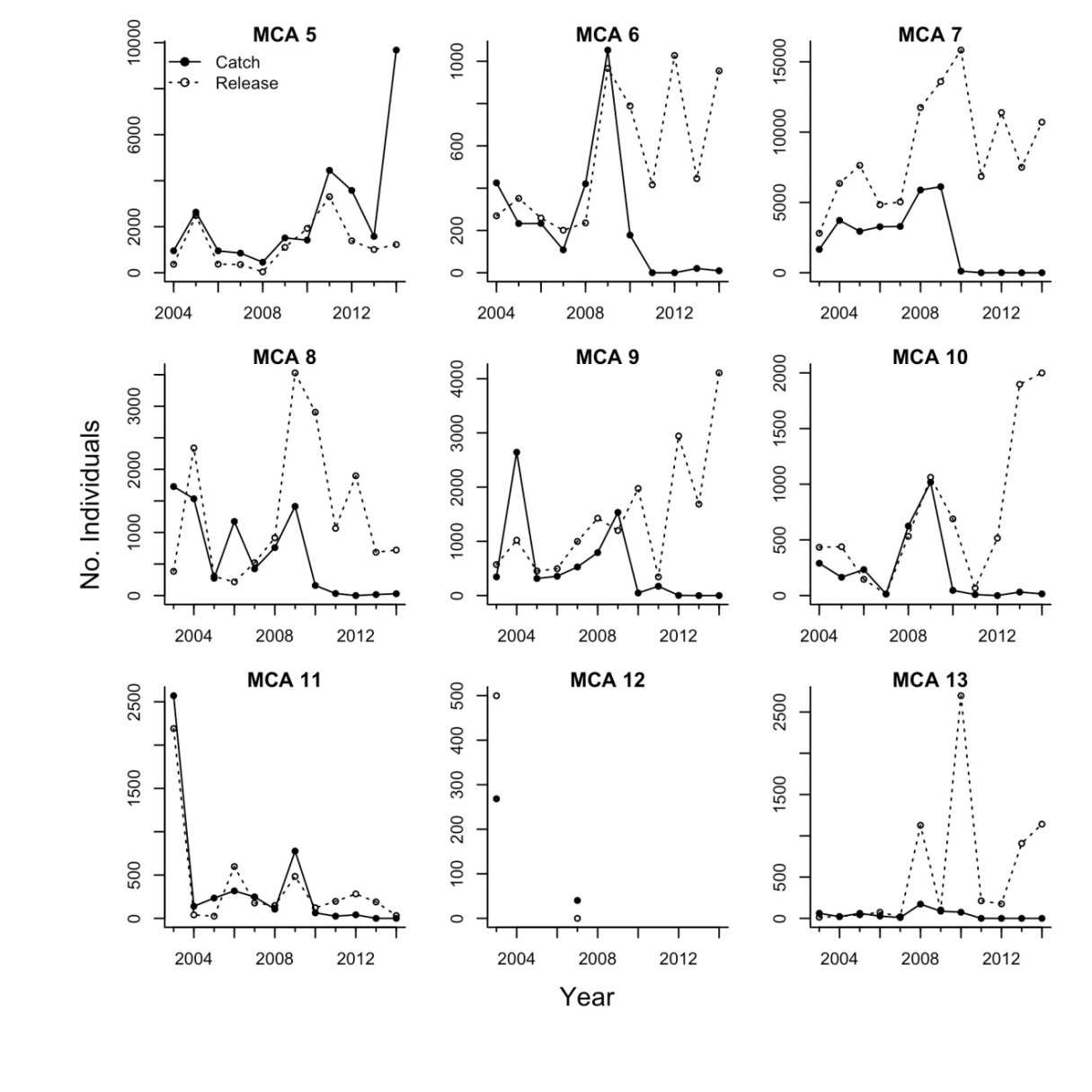


**FIGURE S4** Change in estimated catch (retained catch) and estimated release (catch and release) reported in the WDFW recreational survey from 2003-2008. Catch in MCAs 6-13 from 2010 to 2014 is illicit catch. Some catch, especially of black rockfish, is allowed in MCA 5.

**TABLE S11**  Proportion of the rockfish assemblage represented by bocaccio, canary and yelloweye rockfishes by decade from the WDFW creel (dockside) survey. Top panel includes Marine Conservation Areas (MCAs) 5-13. Bottom section includes only MCAs 6-13. These are the numbers shown in Figure 8.

| Decade | MCA's | Bocaccio | CL_lower_ | CL_upper_ | Canary | CL_lower_ | CL_upper_ | Yelloweye | CL_lower_ | CL_upper_ | Total fish sampled |
| --- | --- | --- | --- | --- | --- | --- | --- | --- | --- | --- | --- |
| 1960's | 5-13 | 0.001 |  |  | 0.044 |  |  | 0.012 |  |  |  |
| 1970's | 5-13 | 0.016 |  |  | 0.061 |  |  | 0.005 |  |  |  |
| 1980's | 5-13 | 0.002 | 0.001 | 0.003 | 0.017 | 0.015 | 0.020 | 0.005 |  |  |  |
| 1990's | 5-13 | 0.001 | 0.000 | 0.003 | 0.004 | 0.002 | 0.008 | 0.001 | 0.000 | 0.004 | 1974 |
| 2000's | 5-13 | 0.000 | 0.000 | 0.001 | 0.004 | 0.003 | 0.005 | 0.002 | 0.001 | 0.003 | 13388 |
| 2010's | 5-13 | 0.001 | 0.000 | 0.001 | 0.003 | 0.002 | 0.004 | 0.004 | 0.003 | 0.004 | 20366 |
| 1960's | 6-13 | 0.001 |  |  | 0.044 |  |  | 0.012 |  |  |  |
| 1970's | 6-13 | 0.016 |  |  | 0.061 |  |  | 0.005 |  |  |  |
| 1980's | 6-13 | 0.002 | 0.001 | 0.003 | 0.017 | 0.015 | 0.020 | 0.005 | 0.004 | 0.006 | 12340 |
| 1990's | 6-13 | 0.001 | 0.000 | 0.003 | 0.004 | 0.002 | 0.008 | 0.001 | 0.000 | 0.004 | 1974 |
| 2000's | 6-13 | 0.000 | 0.000 | 0.001 | 0.004 | 0.003 | 0.005 | 0.001 | 0.001 | 0.002 | 12450 |
| 2010's | 6-13 | 0.001 | 0.000 | 0.001 | 0.001 | 0.001 | 0.002 | 0.000 | 0.000 | 0.001 | 15359 |

**R-code for MARSS simulation**

################################################################

# Code for testing the parameter estimation

# This code was used to produce Figure 2 in the main text.

###############################################################

library(MARSS)

#number of MCAs

mcas=5

#length of segments; matches recreational data

len = c(6,11,6,10,5)

n=sum(len) #num years = 38; same as recreational data

breaks=length(len) #number of segments; 5

#true parameter values for the simulation

mu=-0.02 #2% per year decline

q=.02 #like recreational data estimates

#sim to recreational estimates

#vals for Figure 2 were c(0.13, 0.147, 0.13, 0.09, 0.075)

r=runif(mcas,.06,.2)

#the year when each segment starts

starts = cumsum(c(1,len))[1:length(len)]

#holders

pars=xs=ys=states=c()

#Note this takes a long time

for(sim in 1:1000){

x = cumsum(rnorm(n,mu,sqrt(q))) #true

y = matrix(NA,breaks*mcas,n) #data matrix

# ba and ma are the scalers for the segments and MCAs

# chosen so the true trend is different than the apparent trend

# data appears to show a false increase

ba = rnorm(breaks,seq(0,20,1)[1:breaks],.2) #scale for each break

ma = runif(mcas,-2,2)

for(i in 1:breaks){

for(j in 1:mcas){ #filling in the data matrix

y[j+(i-1)*mcas,starts[i]:(starts[i]+len[i]-1)]=

x[starts[i]:(starts[i]+len[i]-1)]+

ba[i]+ma[j]+runif(1,-2,2)+rnorm(len[i],0,sqrt(r[j]))

}

}

#Set up R to be diagonal with each MCA having its own observation variance

Rm=matrix(list(0),breaks*mcas,breaks*mcas)

diag(Rm)=rep(paste("m",1:mcas,sep=""),breaks)

#BFGS is faster than the default for MARSS()

fit=MARSS(y,model=list(Z=matrix(1,breaks*mcas,1),R=Rm),method="BFGS",silent=TRUE)

#store results

pars=rbind(pars,c(coef(a)$U,coef(a)$Q,coef(a)$R))

states=rbind(states,a$states[1,])

xs=rbind(xs,x)

ys=rbind(ys,y)

}

save(pars,states,xs,ys,fit,n,mu,q,r,breaks,mcas,file="rockfish_sim.Rdata")

pdf(file="Fig_simulation_1.pdf", width=7, height=5)

### Make the plot

leg.inset = c(-0.05,-.05)

par(mfrow=c(2,2),mar=c(5,5,1,2))

#set up an example y

set.seed(111)

x = cumsum(rnorm(n,mu,sqrt(q))) #true

y = matrix(NA,breaks*mcas,n) #data matrix

# ba and ma are the scalers for the segments and MCAs

# did it so the true is totally hidden

# data appears to show a false increase

ba = rnorm(breaks,seq(0,20,1)[1:breaks],.2) #scale for each break

ma = runif(mcas,-2,2)

for(i in 1:breaks){

for(j in 1:mcas){ #filling in the data matrix

y[j+(i-1)*mcas,starts[i]:(starts[i]+len[i]-1)]=

x[starts[i]:(starts[i]+len[i]-1)]+

ba[i]+ma[j]+runif(1,-2,2)+rnorm(len[i],0,sqrt(r[j]))

}

}

# Panel a shows example data

matplot(t(y),pch=rep(1:breaks,mcas),col=rep(1:breaks,mcas),

bty="L",ylab="Survey log abund.\nindex",

xlab="Year",

ylim=c(-3,7))

legend("topleft","a)",bty="n",inset=leg.inset)

abline(v=starts[-1],lty=2)

# Panel b shows x (true state) and estimated state for a case

# where q estimate is non-zero

s=which(pars[,2]>1e-5)[3] # [3] chosen randomly

plot(xs[s,], bty="L",

ylab="Population log abund.\nindex",

xlab="Year",ylim=c(min(xs[s,]),max(xs[s,])+.1));

lines(states[s,]-mean(states[s,]-xs[s,]))

legend("topleft","b)",inset=leg.inset,bty="n")

legend("topright",c("true trend","est trend"),

bty="n",inset=c(0,.1),pch=c(1,-1),lty=c(0,1))

# Panel c shows the histogram of trend estimates

hist(pars[,1],xlab="Long-term trend estimates",

main="",ylim=c(0,350))

abline(v=mu,lwd=2)

text(mu,300,paste("true =\n",mu),pos=2)

text(mu,300,paste("mean est\n =",round(mean(pars[,1]),digits=2)),pos=4)

legend("topleft","c)",bty="n",inset=leg.inset)

# Panel d shows the histogram of process variance estimates

hist(pars[,2],xlab="Process variance estimates",

main="",ylim=c(0,450))

abline(v=q,lwd=2)

text(q,370,paste("true =",q),pos=4)

text(q,310,paste("mean est (>0) =",round(mean(pars[pars[,2]>.0000001,2]),digits=2)),pos=4)

legend("topleft","d)",bty="n",inset=leg.inset)

dev.off()
